# Molecular genetic diversity and differentiation of Nile tilapia (*Oreochromis niloticus*, L. 1758) in East African natural and stocked populations

**DOI:** 10.1186/s12862-020-1583-0

**Published:** 2020-01-30

**Authors:** Papius Dias Tibihika, Manuel Curto, Esayas Alemayehu, Herwig Waidbacher, Charles Masembe, Peter Akoll, Harald Meimberg

**Affiliations:** 1 Institute for Integrative Nature Conservation Research, University of Natural Resources and Life Sciences Vienna (BOKU), Gregor Mendel Straße 33, 1180 Wien, Austria; 2 National Agricultural Research Organization, Kachwekano Zonal Agricultural Research and Development Institute, P.O. Box 421, Kabale, Uganda; 3 Institute for Hydrobiology and Aquatic Ecosystems Management, University of Natural Resources and Life Sciences Vienna (BOKU), Gregor Mendel Straße 33/DG, 1180 Wien, Austria; 4 National Fishery and Aquatic Life Research Centre, P.O. Box 64, Addis Ababa, Sebeta Ethiopia; 5 Department of Zoology, Entomology and Fisheries Sciences-Makerere University Kampala, P. O. Box, 7062 Kampala, Uganda

**Keywords:** Cichlids, Fish translocations, Genetic structure, Gene flow, Bottleneck

## Abstract

**Background:**

The need for enhancing the productivity of fisheries in Africa triggered the introduction of non-native fish, causing dramatic changes to local species. In East Africa, the extensive translocation of Nile tilapia (*Oreochromis niloticus*) is one of the major factors in this respect. Using 40 microsatellite loci with SSR-GBS techniques, we amplified a total of 664 individuals to investigate the genetic structure of *O. niloticus* from East Africa in comparison to Ethiopian and Burkina Faso populations.

**Results:**

All three African regions were characterized by independent gene-pools, however, the Ethiopian population from Lake Tana was genetically more divergent (F_st_ = 2.1) than expected suggesting that it might be a different sub-species. In East Africa, the genetic structure was congruent with both geographical location and anthropogenic activities (Isolation By Distance for East Africa, R^2^ = 0.67 and Uganda, R^2^ = 0.24). *O. niloticus* from Lake Turkana (Kenya) was isolated, while in Uganda, despite populations being rather similar to each other, two main natural catchments were able to be defined. We show that these two groups contributed to the gene-pool of different non-native populations. Moreover, admixture and possible hybridization with other tilapiine species may have contributed to the genetic divergence found in some populations such as Lake Victoria. We detected other factors that might be affecting Nile tilapia genetic variation. For example, most of the populations have gone through a reduction in genetic diversity, which can be a consequence of bottleneck (G-W, < 0.5) caused by overfishing, genetic erosion due to fragmentation or founder effect resulting from stocking activities.

**Conclusions:**

The anthropogenic activities particularly in the East African *O. niloticus* translocations, promoted artificial admixture among Nile Tilapia populations. Translocations may also have triggered hybridization with the native congenerics, which needs to be further studied. These events may contribute to outbreeding depression and hence compromising the sustainability of the species in the region.

## Background

Nile tilapia, *Oreochromis niloticus*, is native to the Levant and African freshwater systems e.g., in the Western part of the continent (e.g., Senegal, Gambia, Niger, Benue, Chad) as well as to many of the East African rivers (e.g. R. Nile) and Rift Valley Lakes like, Albert, Turkana, George, Edward, Tanganyika, Kivu, etc. [1, 2]. Although *O. niloticus* is native to Africa, the cichlid is naturally absent in the world’s largest tropical freshwater body, Lake Victoria and the neighboring Lakes Kyoga and Nabugabo as well as many of the East African satellite lakes [2–5]. These lakes were naturally inhabited by two tilapiine species; *O. variabilis* (Nyasalapia) and *O. esculentus* (Ngege) [2–4]. For more than nine decades, *O. niloticus* has been intentionally dispersed worldwide, in particular for aquaculture and restocking programs [2, 6]. In East Africa, various fish introductions are reported, starting in the 1920s. For example, *O. niloticus,* and other tilapiines e.g. Athi River Tilapia (*Tilapia spilurus nigra,* Günther 1894) as well as Black bass (*Micropterus salmoides*), being initially translocated for enhancing fisheries productivity in water bodies naturally considered as unproductive like the southwestern Uganda high-altitude lakes [7, 8]. A case in point is Lake Bunyonyi which was stocked in the 1920s with individuals of *O. niloticus* from Lake Edward [7]. Similarly, in the 1950s, several tilapiine species were stocked into Lakes Victoria, Nabugabo and Kyoga to counteract the decline of native fish species (*O. variabilis* and *O. esculentus*) [3–5, 9]. The introduced species; *O. niloticus*, *O. leucosticus* (Blue-spotted tilapia)*, Coptodon zillii* (Red-belly tilapia) and *O. melanopleura*, were all suspected to originate from Lake Albert [3, 4, 9, 10]. However, some introductions might have also originated from Lake Edward and Lake Turkana into Lake Victoria basin [2, 4, 8]. Following these introductions, the indigenous fish species in Lakes Victoria, Kyoga, and Nabugabo, significantly declined in the 1980s, coinciding with the dramatic increase in the stock size of the non-native *O. niloticus* [3, 4, 11]. The potential reasons for the declined native fish species (*O. variabilis* and *O. esculentus*) were suspected to a combination of factors including; competition, over fishing, as well as predation pressures from another introduced species, the Nile perch (*Lates niloticus*) [3, 5]. But one additional factor might have been hybridization between the native cichlids (*O. variabilis* and *O. esculentus*) and the introduced tilapiines, particularly *O. niloticus* [4, 9, 11]. Based on these events, the expanded distribution of *O. niloticus* in East Africa complicates the differentiation and identification of genetic units for management and conservation. For example, the population considered as non-native *O. niloticus* in Lakes Victoria and Kyoga might have genetically diverged via admixture and hybridization with the indigenous species [4, 9, 12]. The loss of indigenous *O. mossambicus* due to hybridization with the introduced *O. niloticus* has been reported in South Africa [13]. The situation in East Africa may have worsened with the recent boom of fish hatcheries and aquaculture production systems [14]. In this context, feral populations resulting from escapees might be an additional and serious threat to natural systems.

In nearly the last two decades, the East African countries have been developing measures for fisheries sustainable exploitation through the implementation of co-management strategies [15]. Nonetheless, conservation and management of the already admixed species might not be achieved if the genetic structure of the species in question is not well understood, as the stocks are difficult to define [16]. Therefore, with respect to the East African *O. niloticus,* as the species were potentially affected by various anthropogenic activities, a thorough characterization of the populations at the molecular level might be needed.

Based on the earliest studies, East African *O. niloticus* diversity has been studied using both traditional morphometric methods and molecular markers, which led to contradictory patterns in the species description. For example, using biometrics and counts, seven *O. niloticus* subspecies from African different regions or lakes were described [2]. Nevertheless, this classification was contradicted by subsequent studies using morphometric analyses accompanied by allozyme markers, which indicated that the *O. niloticus* strain from Lake Edward is closely related to that of the lower Nile (Egypt) [17]. Also, other investigations using restriction endonuclease mitochondrial DNA found that *O. niloticus* from Lake Tana is distinct, contrary to the earlier traditional morphometric and meristic analyses [18]. Other earlier molecular genetic studies employing allozymes and restriction fragment length polymorphism (RFLP) of mitochondrial DNA (mtDNA), as well as randomly amplified polymorphic DNA (RAPD) for investigating the demography of *O. niloticus* populations in East Africa, shed some additional light to these incongruences [1, 19, 20]. Some of these studies reported that *O. niloticus* populations from Albert Nile (the Egyptian stretch of River Nile) are distinct from the West African populations, also contrary to earlier morphometric studies [1]. Furthermore, these past investigations based on traditional markers indicated conflicting results amongst. For example, findings from a combination of allozymes and restriction fragment length polymorphism (RFLP) of mtDNA indicated that *O. niloticus* in from Lake Tana is clustered with Lake Edward and the Kenyan Lake Turkana system, which differs from the findings based on restriction endonuclease analysis of mtDNA [1, 18]. These results are inconsistent probably because of the different methodological approaches used that comprise different information content [21–26]. Additionally, the markers used so far have low resolving power to characterize variation within and between populations, and the genetic fingerprinting markers like RAPD cannot discern between homozygotes and heterozygotes [22]. The lack of methodologies with high discriminating power in the past studies, therefore, suggests that the genetic structure patterns of the East African *O. niloticus* are insufficiently documented.

In the present study, we utilize nuclear microsatellite markers, simple sequence repeats (SSRs), to typify the *O. niloticus* in East Africa using next-generation sequencing. SSR loci have been proven robust when investigating the genetic structure of *O. niloticus,* particularly, using SSR genotyping by sequencing (SSR-GBS) [27]. SSR-GBS approaches are useful because they reduce size homoplasy, which is one of the constraints of traditional SSR fragment length analysis [28, 29]. However, SSR-GBS is not without drawbacks [30]. For example, the presence of stutter complicates allele calling for di-nucleotides, null alleles due to mutation on primer binding sites, and it does not recover genomic information hence overestimating events that had a small impact on the gene-pool. Although generally, the use of SSR fragment length analysis can yield information for delineating populations, the recent studies in East Africa that have used this approach on *O. niloticus* were limited to few water bodies in Kenya, with the broader scope of the African Great Lakes missing [31–33]. It is important to conduct a comparative study of various water bodies where *O. niloticus* is present (native and non-native with possible admixture). Such research would provide information on the genetic structure and diversity which would establish a firm base for management and conservation of these cichlids [34].

Here, we explicitly investigate the genetic structure of *O. niloticus*, in East Africa including some populations from Ethiopia and West Africa (Burkina Faso), representing the Sub-Saharan African Great Lakes. We compare natural/native with introduced/non-native *O. niloticus* populations, including other populations from aquaculture systems. With this approach, we investigate the impact of anthropogenic activities, particularly the translocations, on the *O. niloticus*’ gene pool. This is especially important to evaluate the genetic integrity of native stocks. We hypothesized that anthropogenic activities have affected the genetic divergence of *O. niloticus* populations, particularly in environments where the species was introduced. We also predict that the geographical context exhibited by aquatic interconnectivity may influence the genetic homogeneity of cichlid in such environments. We test these hypotheses by answering the following research questions: 1) Does the genetic structure of the East African *O. niloticus* populations differ from those outside the region? 2) To what extent does the genetic structure of the East African *O. niloticus* populations reflect the geography and anthropogenic activities associated with the pathways of the translocation?

## Results

### Variability of SSR loci

In total, 13,530,228 paired reads were produced for genotyping, from which 9,579,578 passed the quality control steps, which were later used for allele calling. Genetic variation results for the 40 SSR loci are presented in the Additional file 1: Table S2. The number of alleles per locus had a mean value of 33.8 ± 20.5, ranging from seven to 84, with a total of 1352 alleles generated across all loci. Overall, 80% of the loci exhibited expected heterozygosity (He) values greater than 0.5. Polymorphic Information Content (PIC) was generally congruent with He, with 78% of loci indicating values of greater than 0.5 (Additional file 1: Table S2).

### Genetic structure

The UPGMA dendrogram showed that all East African populations were more similar to each other than to the other regions (Fig. 2). In this case, the three Ethiopian populations (Hashenge, Ziway, and Chamo) formed the most distant group followed by Burkina Faso and the other Ethiopian water body, Lake Tana. Among the East African natives, the largest separation was between the Kenyan, Lake Turkana, and the Ugandan water bodies. In Uganda, with exception of Lake Victoria, the non-native lakes and fish farms grouped with a native population: the southern Ugandan high-altitude Lakes (Kayumbu and Mulehe) with a group comprised by Lakes George, Edward and Kazinga Chanel; Lake Kyoga populations- and Sindi Farm with River Nile; and Bagena and Rwitabingi farms with Albert. Four subpopulations of Lake Victoria (Gaba, Masese, Kakyanga, Kamuwunga) formed a sister group to the River Nile one. The Lake Victoria subpopulation Sango Bay showed the highest degree of divergence in Uganda.

Neighbor network results showed a similar pattern to the UPGMA dendrogram both at regional and local levels (Fig. 3). In this case, however, Burkina Faso was observed to be closer to the Ugandan populations. In general, network results reflected two Ugandan catchment groups: the George, Kazinga Channel, and Edward group together with the non-native Ugandan highland lakes, and on the other end, Albert and River Nile systems together with the non-native Lake Kyoga and all fish farms. Interestingly Lake Victoria exhibited an intermediate position between both groups with the subpopulation from Sango Bay showing a long branch, suggesting high genetic differentiation. Overall, most of the non-native populations (including farms) showed longer branches than the natives (Fig. 3).

Genetic distance between individuals which was visualized through principal coordinates analysis (PCoA), analysis showed a separation of population groups based on geographic regions (Fig. 4a). Samples formed four groups when analyzed at the regional/country level (Fig. 4a): two groups with individuals from Ethiopia, one with individuals from East Africa, and another intermediate group with samples from both regions. The composition of these groups was clearer when the distance between the native individuals was plotted (Fig. 4b). At this level, Lake Turkana clustered with Burkina Faso, and a division between the three Ethiopian Lakes (Hashenge, Chamo, and Ziway) and Lake Tana was clearly observed. Amongst East African populations, the separation between Lake Turkana and the remaining native populations was evident (Fig. 4b). Individuals found in the Ugandan native populations were divided into two main groups (Fig. 5a). One group was composed of Lake Albert and River Nile individuals while the other by Lake Edward, Kazinga Channel, and Lake George. This division was less evident when individuals from non-native and fish farm populations were included in the analysis (Fig. 5b). Here, some individuals from Sango Bay formed a separate group from the remaining Ugandan individuals. A further group composed of Lake Hashenge individuals was found when only Ethiopian individuals were plotted (Fig. 5c). Substructure within the same lake was only evident for Lakes Victoria and Kyoga (Fig. 6).

The Bayesian analysis with STRUCTURE was portrayed based on the optimal K values. For all populations, the best K was 10, all native populations, K = 7, East African native populations, K = 2, Ugandan native populations, K = 2, and all Ugandan populations including farms, K = 4 (Additional file 1: Figure S2). *O. niloticus* populations from each African region were assigned to different groups (Fig. 7a). Within each region, the same assignments were observed with Lakes Tana and Turkana isolated from the rest of Ethiopians and East African populations, respectively (Fig. 7a). Among the Ugandan native populations, clustering was also congruent with the two water systems, as indicated earlier by both network and PCoA analyses, see Fig. 7b and c. However, there were cases where the non-native populations showed independent clusters from the native. For example, in all analyses, Lake Victoria clusters differed from other populations even when only Ugandan *O. niloticus* were included in the analysis (Fig. 7c). Apparently, admixture was more evident amongst the East African populations but mostly detected when only non-native populations were considered (Fig. 7c).

### Gene flow between population

Results from recent migration rates estimated with BayesAss indicated that Lakes Kyoga and George were the main sources of migration (Fig. 8), with values for other populations generally falling below (< 2%). Noticeable gene flow was from Lakes Kyoga to Victoria and George to Edward (27%), Kyoga to Albert (25%), Kyoga to Bagena farm (23%), Kyoga to Sindi farm, River Nile and Rwitabingi farm (22%), George to Kazinga Channel (21%) and finally George to Mulehe (20.4%) (Fig. 8). Migration rates estimated through Genalex were congruent with BayesAss, but with the difference that the *O. niloticus* population from Lake Victoria was also a source of migrants (Additional file 1: Table S3).

### Genetic differentiation, diversity, and isolation by distance

Genetic differentiation of *O. niloticus* was consistent with the STRUCTURE results. For instance, the F_st_ values clearly demonstrated that the East African *O. niloticus* populations are genetically distant from the Ethiopian and West African populations (Fig. 9a). Despite *O. niloticus* populations from River Nile and Lake Kyoga showing relatively high F_st_ values, results from the East African populations generally showed low genetic differentiation. Also, the East African *O. niloticus* populations were genetically more diverse when compared to either Ethiopian or Burkina Faso (Fig. 9b-d). Based on all statistics, the non-native Lake Victoria and native Lake Turkana *O. niloticus* populations were the most genetically diverse. On the other hand, Lake Kyoga and River Nile *O. niloticus* populations were consistently the least diverse even when investigated at the subpopulation level (additional file 1: Figure S4).

Results from the Garza-Williamson index (G-W), generally indicated that nearly all of the studied populations went through a bottleneck, apart from the Ethiopian Lake Tana (Fig. 10a). In the analysis, only Lake Tana exhibited G-W values > 0.5 (0.56 ± 0.44). Regarding population genetic diversity, however, Lakes Victoria and Turkana showed the highest number of private alleles (Fig. 10b).

When we partitioned Lake Victoria to assess the genetic diversity patterns within the water body, generally one sub-population was distinguished from the others (Fig. 11). Sango Bay, in particular, was isolated based on F_st_ values, and consistently exhibited higher genetic diversity indices (Na, He and Ar) (Fig. 11).

Mantel tests for isolation by distance (IBD) across all samples showed a positive correlation between geographical and genetic distance (R^2^ = 0.30) (Fig. 12a). However, the strong correlation (R^2^ = 0.67) between the populations was only found when Burkina Faso was excluded from the analysis (Fig. 12b). The genetic differentiation between the East African and the Ethiopian populations appears to inflate this correlation. Similarly, a strong IBD was also found amongst East African populations (Fig. 12c), which was not the case when only Ugandan populations (excluding Turkana) were considered (Fig. 12d).

## Discussion

Fisheries and fishery products are vital in the developing world but heavily threatened through various anthropogenic activities which may compromise the continuity of the resources [35]. One aspect of the anthropogenic threats is the change or alteration of the natural genetic structure of fish stocks through admixture [36, 37]. Understanding the admixture of stocks is only possible if the source populations can be differentiated using genetic markers. We show the importance of SSR-GBS for a deeper understanding of population dynamics, in particular, the East African *O. niloticus*, towards the alignment of management and conservation strategies. In this study, we investigated the phylogeographical patterns and we found large differences between lakes (e. g. Lake Tana) and also differences between natural water catchments that allow populations to be identified. Here, we discuss the current state of *O. niloticus* in reference to phylogeographical patterns and anthropogenic activities.

### Phylogeography of east African *O. niloticus*

In all analyses, we found a clear differentiation among all three African regions included in this study (East Africa, Burkina Faso, and Ethiopia), indicating a low degree of connectivity amidst them and highlighting the high level of differentiation between regions. Lake Tana was completely distinct from the remaining populations. This applies not only to the Ethiopian populations but also to the East African ones. So, the genetic distance in Ethiopia is higher than between the East African and West Africa populations, indicating a divergence higher than we would expect within a species. These results are consistent with previous genetic reports [18], but not the findings of the subspecies treatment based on the traditional morphometric and meristics [2]. This high level of differentiation argues for a revision of the species delimitation for these populations.

Lake Tana lies in the Ethiopian mountains and is isolated from the Lakes in the Rift valley [38]. This might explain the high degree of differentiation of this lake because of the lack of connectivity and divergent ecological conditions. Contrary, Lake Hashenge which is also in the Ethiopian mountains is related to the Rift Valley lakes. Lake Hashenge is reported to have been stocked with *O. niloticus* following mass mortalities of the native species [39]. The native status of this lake is unclear since it could have been restocked with *O. niloticus* that originated from the Rift Valley Lakes. Besides that, we see a slight differentiation in PCoA between Lake Hashenge and the Rift Valley Lakes in Ethiopia, which may reflect an unsampled source of stocking or differentiation accumulated because of the high degree of isolation of the lake.

In East Africa, genetic structure reflected different catchments. The population from Lake Turkana was genetically distinct from the Ugandan populations which is expected given its high geographical isolation [40]. Our findings concur with the previous works that treated the Turkana population as a different subspecies (*O. vulcani*) [2]. The high diversity and number of private alleles found in Lake Turkana can be a consequence of this isolation. The East African arid, Lake Turkana, naturally is also characterized by a remarkable genetic diversity. One factor might be introgression perhaps from anthropogenic activities or influx of gene flow from River Omo (Ethiopia). However, this is not clear and a better sampling from the region needs to be included to evaluate the extent of the observed current genetic structure of the population.

In Uganda, despite the high degree of connectivity and proximity between the water bodies, *O. niloticus* populations were clearly structured. These reflected three main groups: 1) (Lakes George and Edward, as well as Kazinga Channel, 2) Lake Albert, River Nile, and Kyoga and 3) Lake Victoria system. The 2nd and 3rd groups are discussed in more detail under anthropogenic activities subsection. The 1st group, Lakes George and Edward are connected via the Kazinga channel which also explains the high natural migration rates between these populations. The different genetic structure between the western Rift Valley Lakes (Edward-George-Kazinga Channel and Albert) was conserved despite being connected through River Semliki that flows from Lake Edward and Albert [41]. The strong rapids and falls present in this river [41, 42], might constitute a strong barrier to gene flow, which maintains these systems apart. These findings are congruent with recent work on *O. niloticus* geometric morphometrics [43] but do not concur with past studies [2, 20]. This incongruity might be associated with different methodological approaches utilized between the earliest studies and the current one. For example, using morphometric and meristics methods, *O. niloticus* from the Edward-George system and Albert was treated as one subspecies; *O. niloticus eduardianus* [2]. However, inference from traditional morphometrics are weak due to the lack of informative characters [18]. Similarly, while we used SSR-GBS techniques, [20] employed random amplified polymorphic DNA (RAPD) markers, which due to their dominance genotypic nature, provide only part of the information content [22].

### Anthropogenic activities-fish translocations

In East Africa, we know that *O. niloticus* was introduced into several water bodies through stocking activities. We were able to genetically track these translocation events to both non-native water bodies and fish farms. All genetic structure analyses and migration rates showed that the two Ugandan groups (the George-Edward complex and Lake Albert) contributed to the stocking of different water bodies. *O. niloticus* from the southwestern Ugandan high-altitude Lakes; Mulehe and Kayumbu, originated from the Western Rift Valley Lakes – Edward and George. For the 2nd group, Lake Kyoga and River Nile (Victoria Nile) are genetically similar to Lake Albert, suggesting that, the latter population might have contributed genes to the gene-pool of the former systems. Although Lake Kyoga is connected to Lake Albert via River Nile, their genetic similarity is unlikely related to the consequence of natural migration via water flow. The main reason here is the natural occurrence of Murchison Falls on the River Nile that acts as a barrier between the systems [3, 41]. For this matter, the genetic similarity between River Nile, Lakes Kyoga, and Albert populations may have resulted in stocking regimes using the latter as source [3].

Fish farms seem to have sourced fish seed from multiple populations, resulting in admixed stocks. Our results show that Lakes Albert, and Kyoga, as well as River Nile, contributed to the gene pool of the farmed populations (Figs. 3, 8 & 7c). Based on genetic distance, Lake Albert was the main contributor to Rwitabingi and Bagena farms while Kyoga to Sindi farm. However, we also observed a high amount of gene flow from Kyoga to Rwitabingi and all these farms appeared to be admixed with other populations including Lake Victoria. Apart from farms, evidence of admixture was probable in the East African natural populations, which seems to have been promoted by anthropogenic activities [3, 9]. This is supported by the fact that when non-native populations were unconsidered in the STRUCTURE and PCoA analyses, signals of admixture were minimal, and clear genetic structure assignments could be observed. In East African, admixture in *O. niloticus* populations may stem from three main processes: 1) translocation from multiple sources into the non-native water bodies, 2) back translocation from non-native to native populations, and 3) hybridization of *O. niloticus* with congeneric species promoted by translocations.

The first and third processes may explain partly the genetic variation found in the 3rd group; Lake Victoria (see above the three Ugandan groups). Although *O. niloticus* in Lake Victoria is generally isolated, based on the distance neighbor Network tree (Fig. 3), the population occupied an intermediate position between the above described; 1st and 2nd, Ugandan groups. Thus, it is clearly possible that multiple stockings might have contributed to the gene-pool indicated by the Lake Victoria population. For example, [2] suggests that introductions into Lake Victoria may have originated from Lake Edward, with other authors suggesting multiple sources [4, 5, 12, 44], which support our results. The highly diverse and differentiated gene-pool in Lake Victoria could have originated from the admixture of several lineages due to multiple sources.

On the other hand, possible hybridization of the introduced *O. niloticus* with the indigenous relative species (*O. variabilis* and *O. esculentus*) in Lake Victoria may explain some of the genetic variation patterns found in this lake. First, this lake together with Turkana showed values of private alleles up to four times higher than the remain populations. This genetic variation could have originated from introgression by species that have not been included in the analysis. Similarly, the probable hybridization may explain the high genetic diversity and divergent gene-pool detected in the system. Within Lake Victoria, the Sango Bay subpopulation appears to be an extreme case from this by showing the highest degree of genetic divergence. Remarkable genetic differentiation in Sango Bay was noticed only when compared with the remaining subpopulations within the lake, but also with the other East African populations. In this case, during the boom of the *O. niloticus* population in Lake Victoria [3–5, 45], a larger portion of the native species’ genetic materials may have been introduced into *O. niloticus* gene-pool. This is just a hypothesis since, in this study, we cannot directly test for hybridization because we did not include samples of *O. niloticus* congenerics. However, hybridization involving *O. niloticus* and other tilapiines has been reported to be relatively frequent and it needs to be considered [9, 33, 46, 47].

If admixture/hybridization shaped the gene-pool of Lake Victoria, it may have adaptive consequences and compromise the sustainability of *O. niloticus*. Although hybridization may lead to heterosis/hybrid vigor [48, 49], admixture is usually reported to have negative consequences [37, 50]. Introgression can contribute to outbreeding depression either by the introduction of maladaptive alleles or through the dilution of alleles important for local adaptation [51]. In more drastic scenarios, hybridization can result in genomic incompatibilities contributing to a fast reduction of population fitness [51]. Alternatively, the hybrids may potentially exhibit more fitness and subsequently extirpate the parental lines [46]. The observed genetic structure of *O. niloticus* populations in Lake Victoria was unexpected and has not been reported before, which calls for further investigations for taxonomic recognition.

Evidence for the second process of admixture was only found in Lake Albert. In the structure analysis, this population showed admixture with Lake Kyoga. We also found significant migrations from Lake Kyoga to Lake Albert. These results indicated that admixture with respect to translocations not only contributes to non-native populations but also to native ones. The sequence of gene flow from Lake Kyoga to Albert is not clear as none of the previous reports have indicated this. However, it is likely that aquaculture activities might be contributing to the observed gene flow between Lakes Kyoga and Albert.

### Anthropogenic activities-consequences of overfishing

Some water bodies, especially Lake Kyoga and River Nile showed low genetic variability and evidence of bottleneck with respect to G-W estimations. Given the recent stocking of these water bodies, this pattern may be explained by the founder effects. Nevertheless other anthropogenic activities need to be considered as well. High loss of genetic diversity among populations, particularly, in fishes has been attributed to over-exploitation [52]. This might be the case for the L. Kyoga population. For example, although *O. niloticus* boosted the capture fisheries in the Lake Victoria basin (Lakes Victoria and Kyoga) following introductions, the species was subsequently overexploited between the 1970s and 80s [53, 54]. This was reflected in the dramatic decline of the stock sizes and increased fecundity, which are clear indicators of overfishing [54]. The low diversity in River Nile could be linked to low gene-flow connectivity with other water bodies due to hydro-electric power dams that have been constructed along the river (the upper Nile of the Ugandan side), which increases the effect of genetic drift. However, this needs to be assessed in further analyses, especially when additional samples are collected in sections of the lower Nile (below Murchison falls), where apparently there are no dams.

#### Implications for management and outlook

Overall, we found evidence that anthropogenic activities affected the gene-pool of the East African *O. niloticus*. The main consequence might have been admixture and potentially hybridization between different stocks and species respectively. In the long term, this may have negative effects on population fitness due to outbreeding depression and genetic swamping. Thus, management measures should inhibit any form of unauthorized spread of fish in the aquatic ecosystems. The Western or Albertine Rift Valley lakes (Edward-George) may be ideal broodstock sources for subsequent breeding programs and aquaculture, as these systems seem not to be admixed. To avoid an influx of feral populations, a proper environmental impact assessment should be prioritized before implementation. Genetic diversity might also have been affected by overfishing and the construction of hydropower dams, which should also be taken into consideration in future management options.

## Conclusions

Our results were congruent with the hypothesis that anthropogenic activities affected the genetic structure of *O. niloticus* populations in East Africa. The genetic variation of some populations, especially from Lake Victoria, corresponded with possible hybridization of *O. niloticus* with native congeneric species, which may have been mediated by anthropogenic activities. This study also contributed to the knowledge of *O. niloticus* phylogeography in East Africa. In this case, we found several new genetic groups such as the populations from Lake Tana, Victoria and the two natural catchments in Uganda. Some of these may require further taxonomic exploration. Additionally, we show that gene-flow among the East African *O. niloticus* populations was not entirely from native to non-native environments, but also from non-native to native environments likely through aquaculture and restocking programs. Moreover, this study shows the importance of molecular markers, in particular, the use of SSR-GBS in cataloging populations. Further studies should include *O. niloticus* samples from other regions such as the lower Nile (below Murchison Falls), Lake Kivu (Rwanda), Tanganyika and Baringo as well as the congenerics for a more comprehensive picture.

## Methods

### Sampling/study areas

**Fig. 1:**
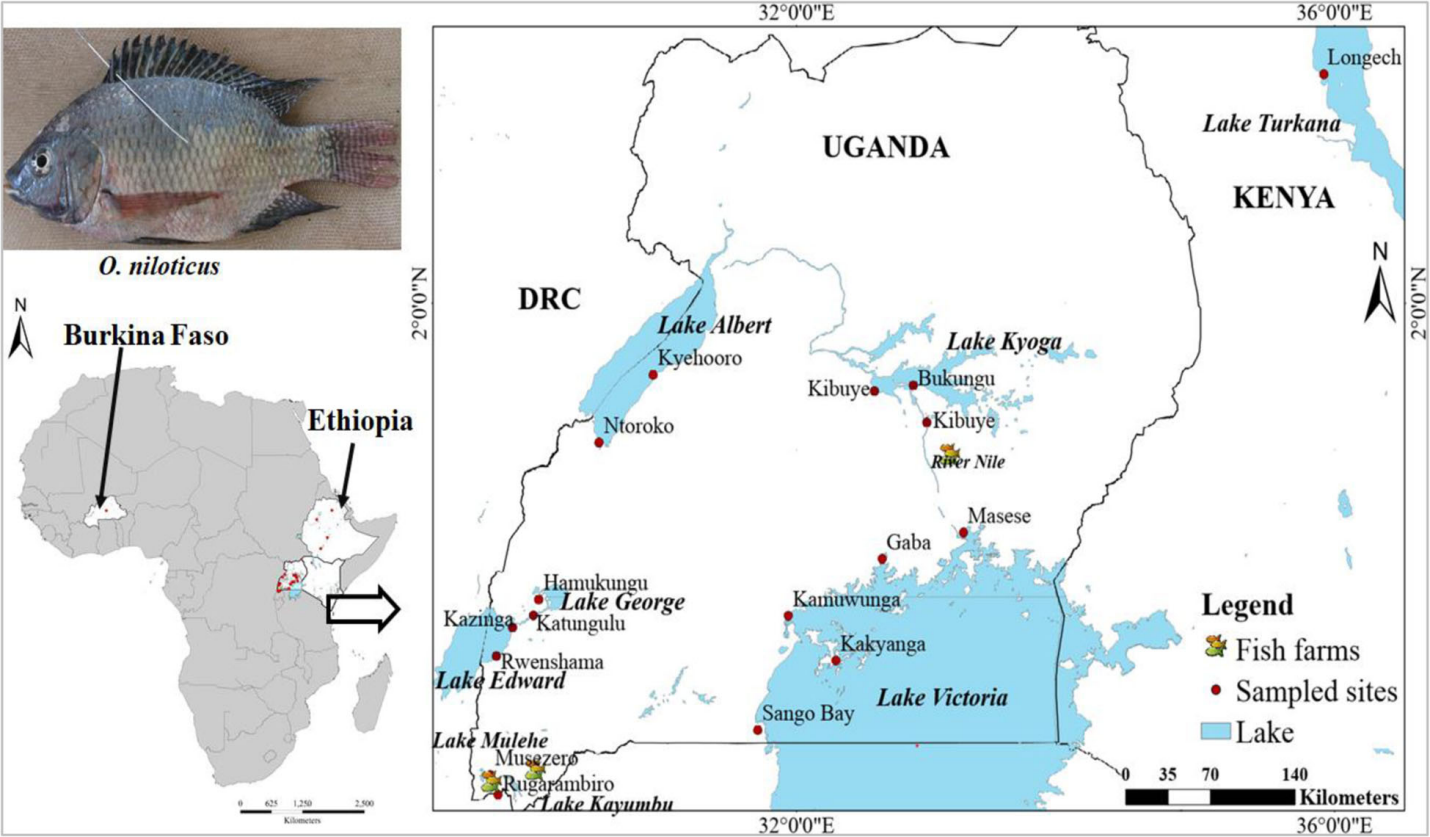
Illustration of sample collection and sources in the African Great Lakes region; East Africa (Uganda and Kenya), Ethiopia and Burkina Faso; modified from our previous work [43]

We collected *O. niloticus* specimens from three water body types: a) those where *O. niloticus* is native, b) where introduced, and c) from fish farms (Fig. 1), following our earlier sampling design [43]. Most samples were collected by local fishermen using gill nets set overnight. At Lake Turkana, a seine net was utilized. From Ethiopia and Burkina Faso, four and one native populations were sampled, respectively. Considering the large extent of Lake Victoria and multiple *O. niloticus* introductions into the world’s largest tropical freshwater body, we sampled five locations to assess possible genetic heterogeneity within the system (Fig. 1). Similarly, in other relatively large lakes like Lake Edward, Kyoga, and Albert, we sampled two locations each for subsequent subpopulation analyses (Table 1). A total of 664 samples were collected from 18 water bodies during several field excursions in 2016. From every single fish, a muscle tissue sample (approx. 30 mg) was extracted from the dorsal region, preserved in absolute ethanol contained in 2 ml Eppendorf tubes and later stored in a freezer until genotyping at the Institute for Integrative Nature Conservation Research-University of Natural Resources and Applied Life Sciences Vienna (BOKU), Austria. Sampling was conducted in collaboration with respective authorities per region and therefore no special permission was required. In all cases, the fish were already dead when obtained from the fishermen, therefore no special treatment for the animals was administered in the process. As contamination of the specimens was not likely during sampling with gill nets, great care and attention were provided for during seining on Lake Turkana. The non-native and farm populations were only sampled in Uganda. Here, we refer to the non-native populations like those found in the high-altitude satellite lakes of south-western Uganda (Lakes Mulehe and Kayumbu) as well as in lower altitude lakes (Lake Victoria and Kyoga) [43]. The three sampled fish farms include; Rwitabingi (located near River Nile and Lake Kyoga), Bagena and Sindi from South-western Uganda. The rest of the populations are regarded as native (Fig. 1; Table 1).

**Table 1:** Details of the sampling sites and the total number of individuals collected per water body and location/site. The indigenous *O. niloticus* populations, are also herein referred to as natives and introduced, non-natives and farms are the pond culture systems

Lakes/River	Sample Site	Local name	No.	Country	Pop. nature	Coordinates		Elev. (m)
Albert	Ntoroko	Ngege	21	Uganda	Indigenous	N.01.05206^0^	E030.53464^0^	618
Albert	Kyehooro	Ngege	16	Uganda	Indigenous	N.01.50990^0^	E030.93610^0^	615
Edward	Rwenshama	Ngege	27	Uganda	Indigenous	S.00.40459^0^	E029.77283^0^	908
Edward	Kazinga	Ngege	22	Uganda	Indigenous	S.00.20783^0^	E029.89252^0^	914
George	Hamukungu	Ngege	35	Uganda	Indigenous	S.00.01739^0^	E030.08698^0^	916
Kazinga Ch	Katungulu	Ngege	21	Uganda	Indigenous	S.00.12541^0^	E030.04744^0^	915
R. Nile (VN)	Kibuye	Ngege	24	Uganda	Indigenous^a^	N.01.18734^0^	E032.96865^0^	1062
Kyoga	Kibuye	Ngege	44	Uganda	Introduced	N.01.40028^0^	E032.57949^0^	1034
Kyoga	Bukungu	Ngege	22	Uganda	Introduced	N.01.43873^0^	E032.86809^0^	1045
Victoria	Kakyanga	Ngege	30	Uganda	Introduced	N.00.18079^0^	E032.29332^0^	1136
Victoria	Gaba	Ngege	26	Uganda	Introduced	N.00.25819^0^	E032.63727^0^	1146
Victoria	Masese	Ngege	23	Uganda	Introduced	N.00.43650^0^	E033.24081^0^	1136
Victoria	Sango Bay	Ngege	25	Uganda	Introduced	N.00.86772^0^	E031.71332^0^	1129
Victoria	Kamuwunga	Ngege	25	Uganda	Introduced	S.00.12747^0^	E031.93999^0^	1139
Mulehe	kisoro	Ngege	25	Uganda	Introduced	S.01.21345^0^	E029.72668^0^	1801
Kayumbu	Kisoro	Ngege	30	Uganda	Introduced	S.01.34679^0^	E029.78446^0^	1901
Rwitabingi	Kamuli	Ngege	29	Uganda	Fish farm	N.00.97116^0^	E033.13924^0^	1069
Bagena	Kisoro	Ngege	31	Uganda	Fish farm	S.01.25617^0^	E029.73622^0^	1857
Sindi	Kabale	Ngege	25	Uganda	Fish farm	S.01.17578^0^	E030.06198^0^	1733
Turkana	Longech	Ngege/Sato	35	Kenya	Indigenous	N.03.55617^0^	E035.91599^0^	364
Ziway	Ziway	Koroso	27	Ethiopia	Indigenous	N8.00730866	E38.8413922	1636
Tana	Tana	Koroso	32	Ethiopia	Indigenous	N12.0266003	E37.3036142	1831
Hashenge	Hashenge	Koroso	26	Ethiopia	Indigenous^a^	N12.5746028	E39.4966667	2443
Chamo	Chamo	Koroso	25	Ethiopia	Indigenous	N5.82128333	E37.5747222	1110
Loumbila	Loumbila	Tegr-pere	18	Burkina Fs	Indigenous^a^	12.5142528″N	01.3972222”w	276

Kazinga *Ch* Kazinga Channel, *VN* Victoria Nile, Burkina *Fs* Burkina Faso, and Population nature with the asterisk symbol (^a^) implies that the population might not be indigenous, *No*. Number of samples, *Pop*. Population, and *Elev*. Elevation.

**Fig. 2:**
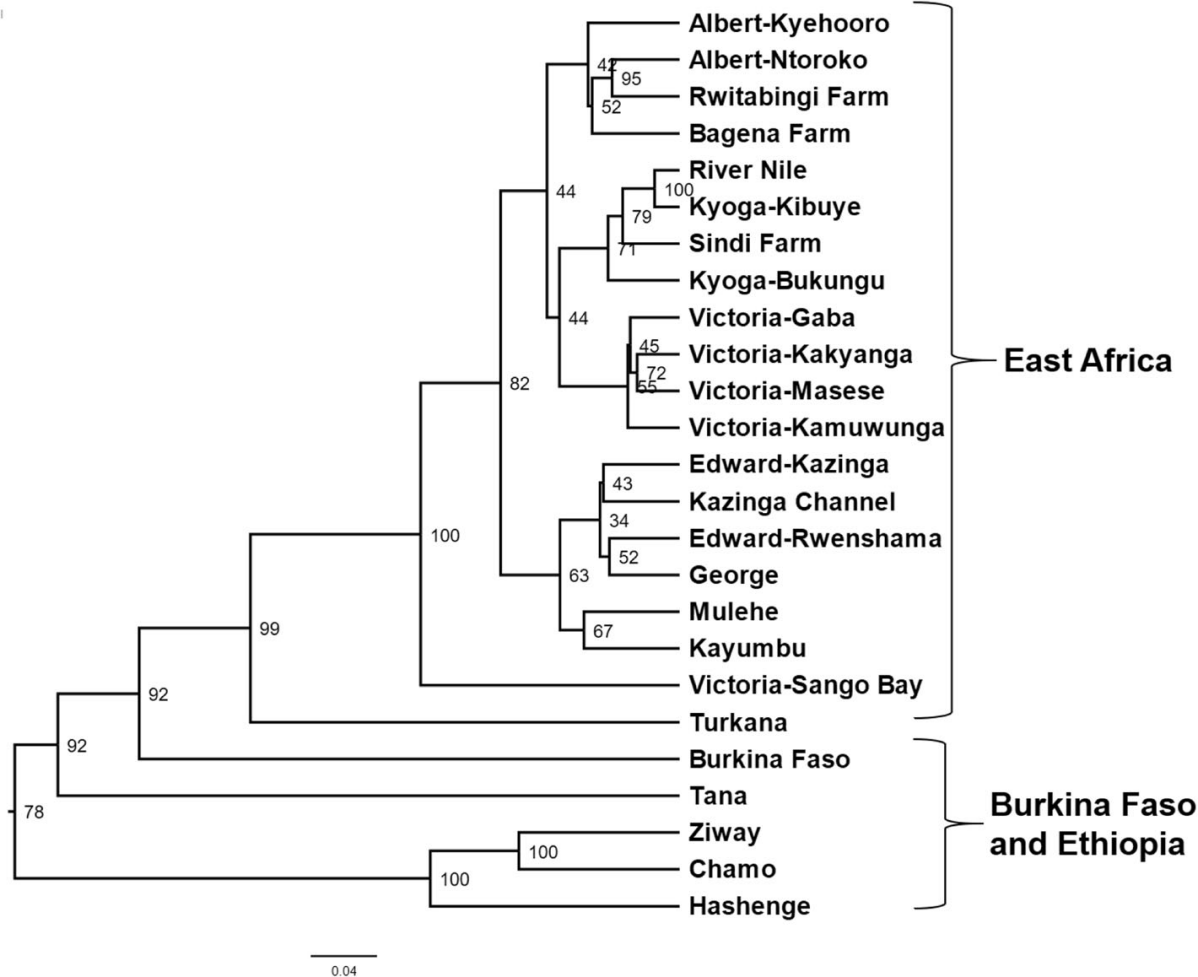
Genetic structure of *O. niloticus* populations based on UPGMA dendrogram. Node values correspond to bootstrap values

**Fig. 3:**
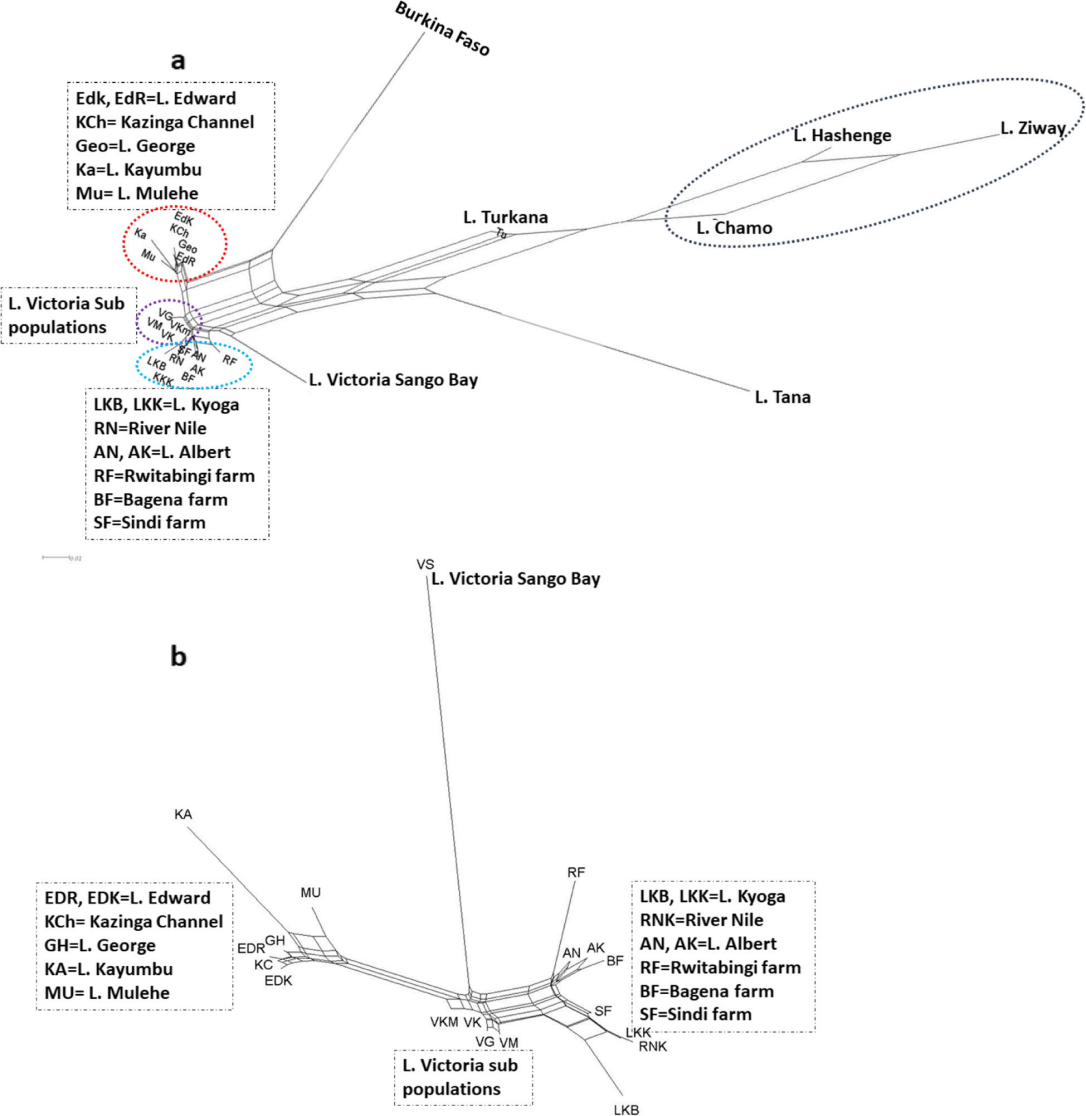
Genetic structure based on unrooted network tree illustrating population relationships based on genetic distance. **a** represents a network tree for all the populations and **b** for only the Ugandan populations. Dotted oval and rectangular shapes depict closely related genetic groups

**Fig. 4:**
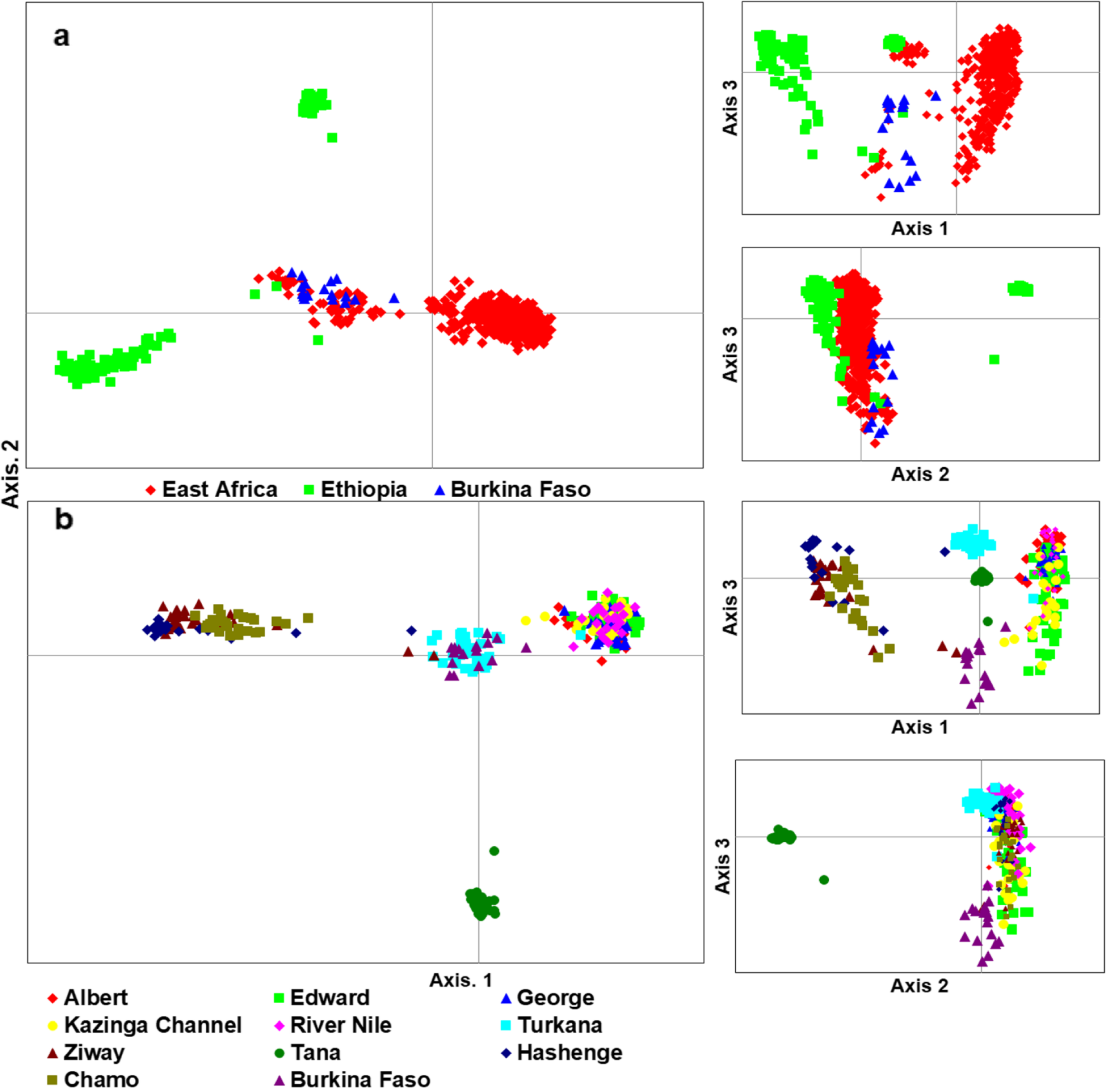
Genetic scatter plots of *O. niloticus* exhibited by Principal Coordinate Analysis (PCoA). **a** populations per region, **b** all indigenous populations. PCoA was constructed with respect to unbiased Nei’s genetic distance among individuals

**Fig. 5:**
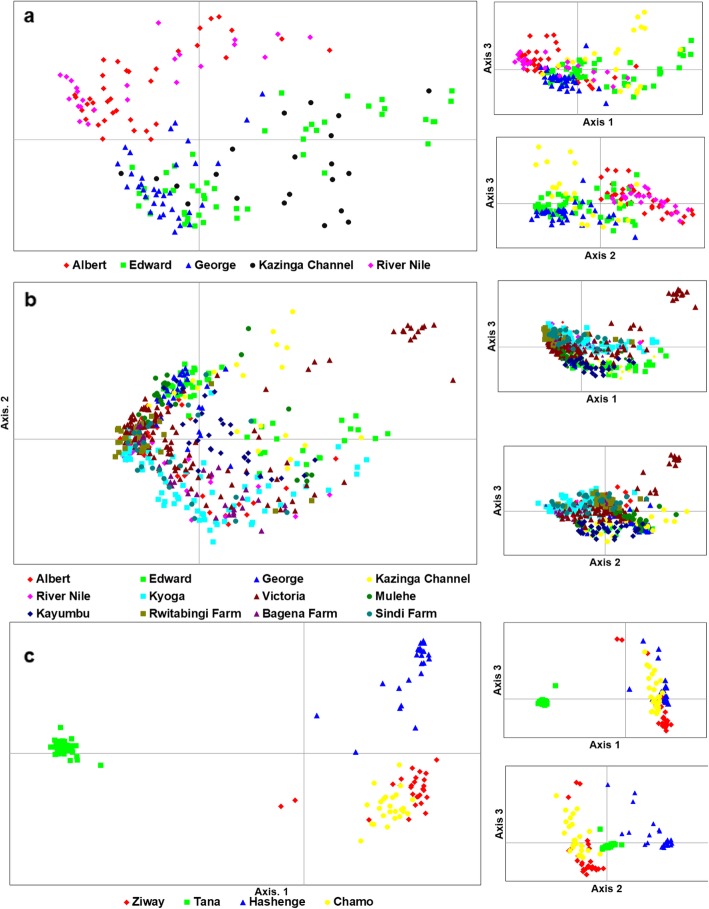
Genetic scatter plots of *O. niloticus* based-on Principal Coordinate Analysis (PCoA). **a** Ugandan native populations, **b** all Ugandan populations including non-natives, natives, and farms, and **c** all Ethiopian populations

**Fig. 6:**
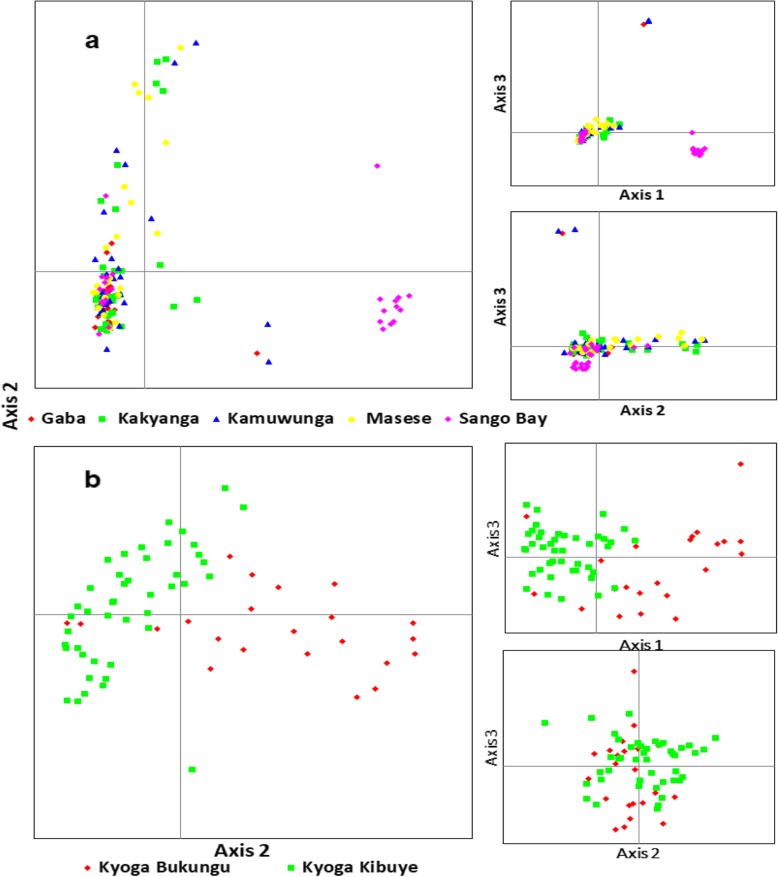
Genetic scatter plots of *O. niloticus* exhibited by PCoA within Lakes Victoria (**a**) and Kyoga (**b**) populations

**Fig. 7:**
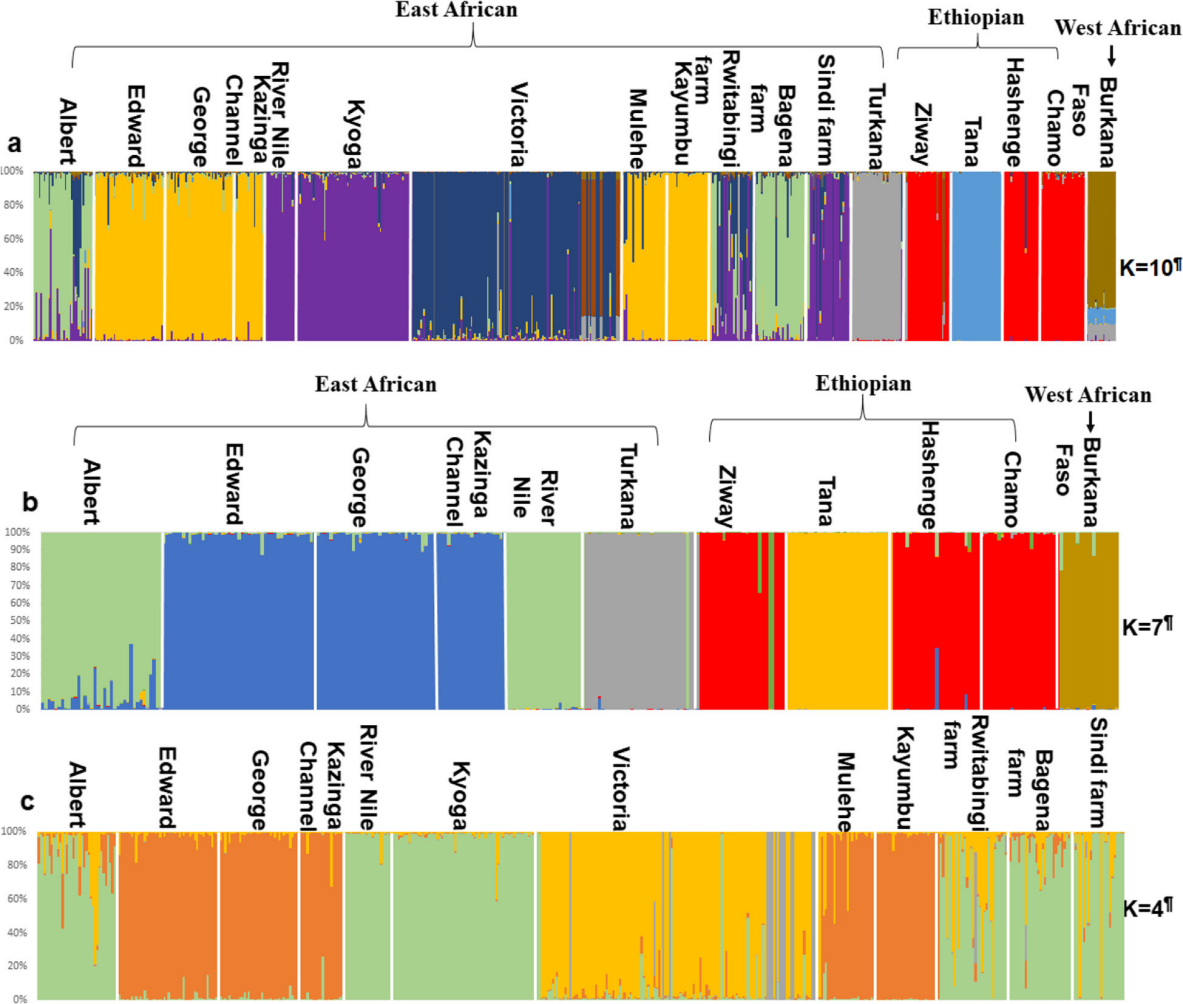
Bayesian clustering for genetic assignments of *O. niloticus* populations. **a** represents all populations, **b** all indigenous populations, and **c** all Ugandan populations including indigenous, non-indigenous and farms. Ks with a superscript symbol (¶) indicates the optimal K values based on STRUCTURE HARVESTER analyses

**Fig. 8:**
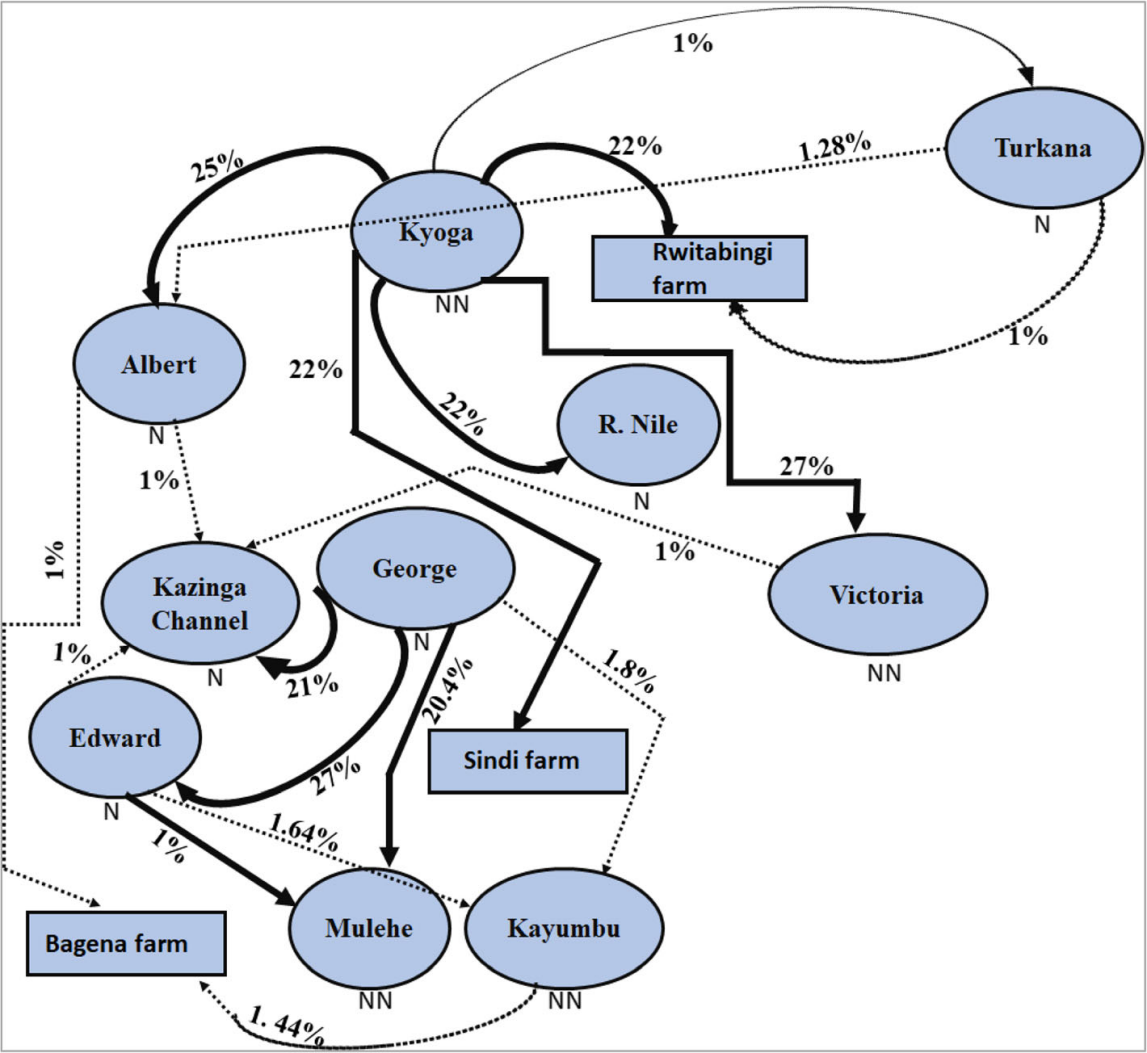
Bayesian inference of recent migratory rates for the 13 East African *O. niloticus* populations. Oval light blue and rectangular light-blue shapes indicate natural and farm populations, respectively. The arrows contain percentage values showing the direction and magnitude of gene flow. Darker and thick arrows represent stronger gene flow, while thin, dotted arrows indicate weaker gene flow. Native and non-native populations are indicated by the letters, “N” and “NN”, respectively. This analysis is based on BayesAss program and for GenAlex program, see the Additional file 1: Table S3

**Fig. 9:**
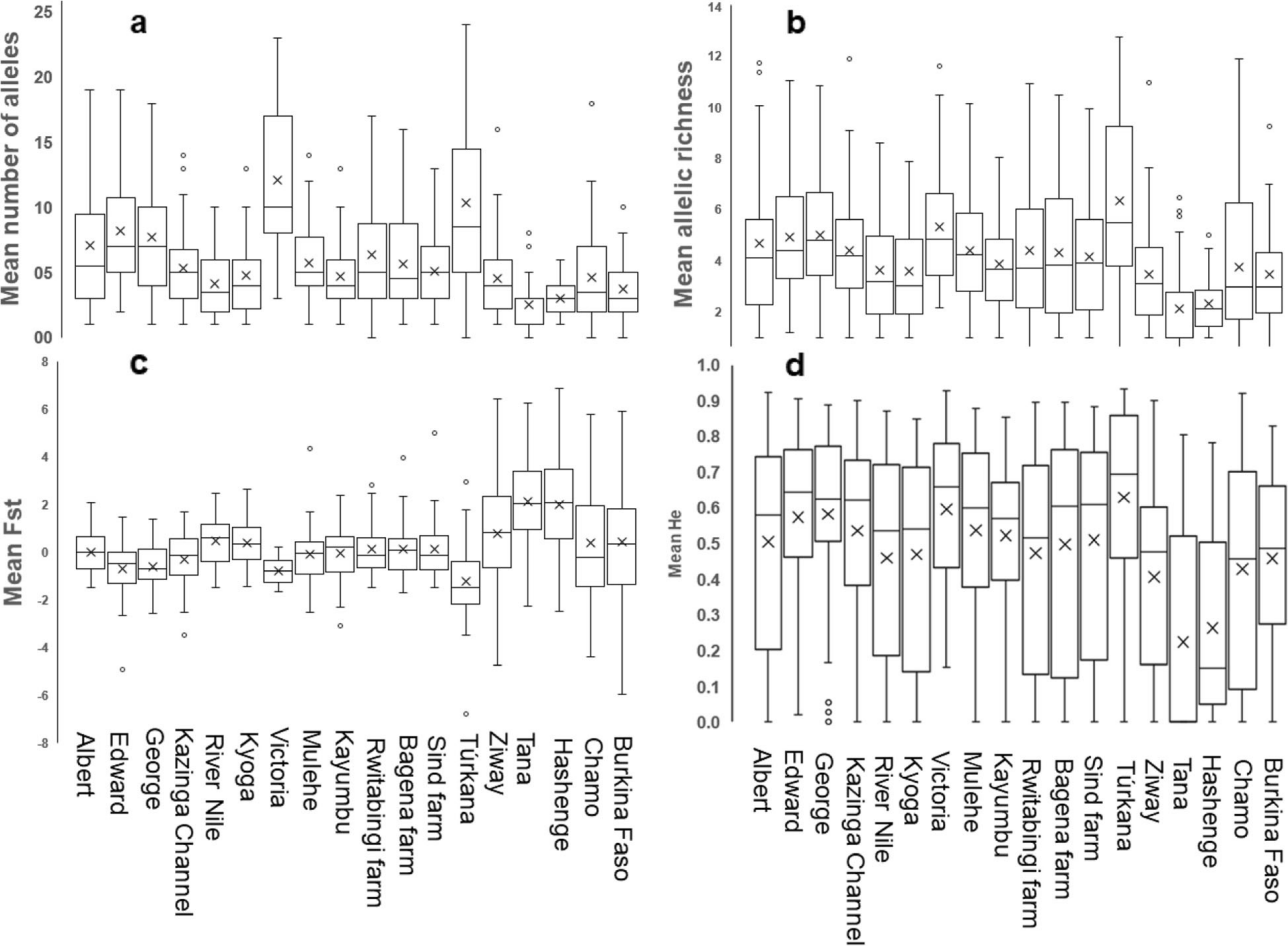
Genetic diversity and differentiation indices. **a** number of alleles, **b** allelic richness, **c** fixation index (F_st_) and **d** expected heterozygosity

**Fig. 10:**
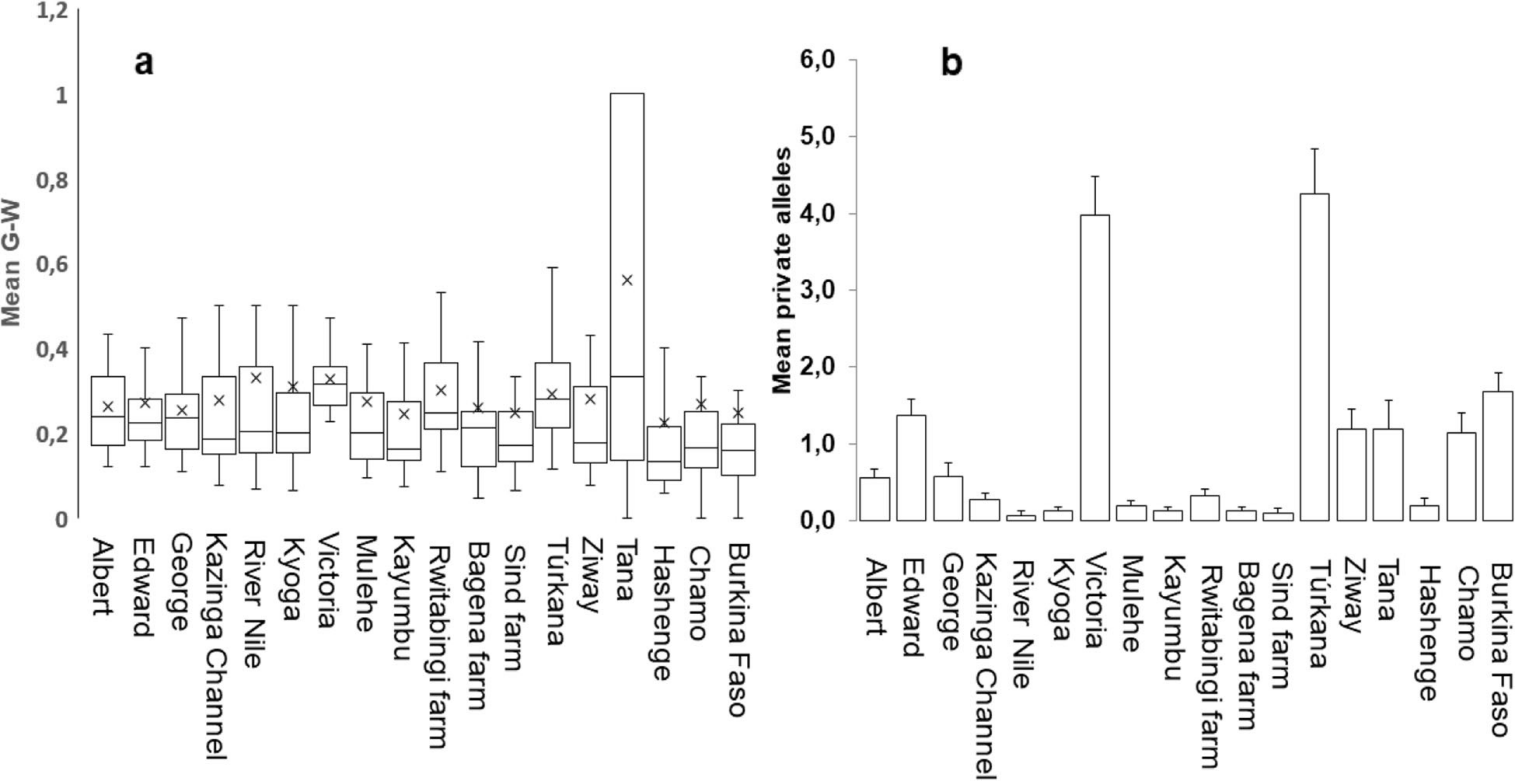
Estimations of population bottleneck derived from Garza-Williamson Index (G-W) (**a**) and measure of genetic diversity based on private alleles (**b**)

**Fig. 11:**
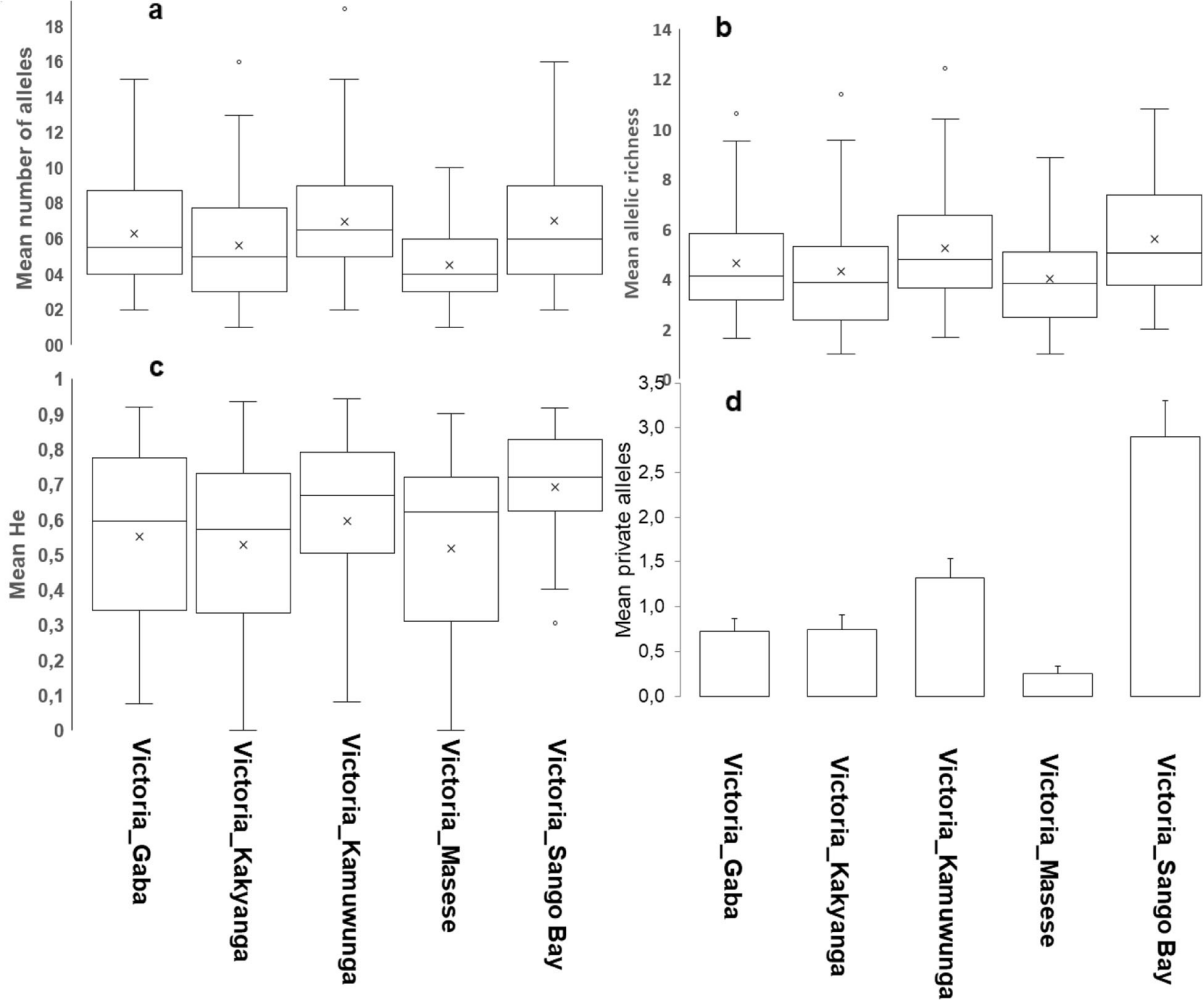
Genetic diversity of Lake Victoria within the population. **a** number of alleles, **b** allelic richness **c** expected heterozygosity and **d** private alleles

**Fig. 12:**
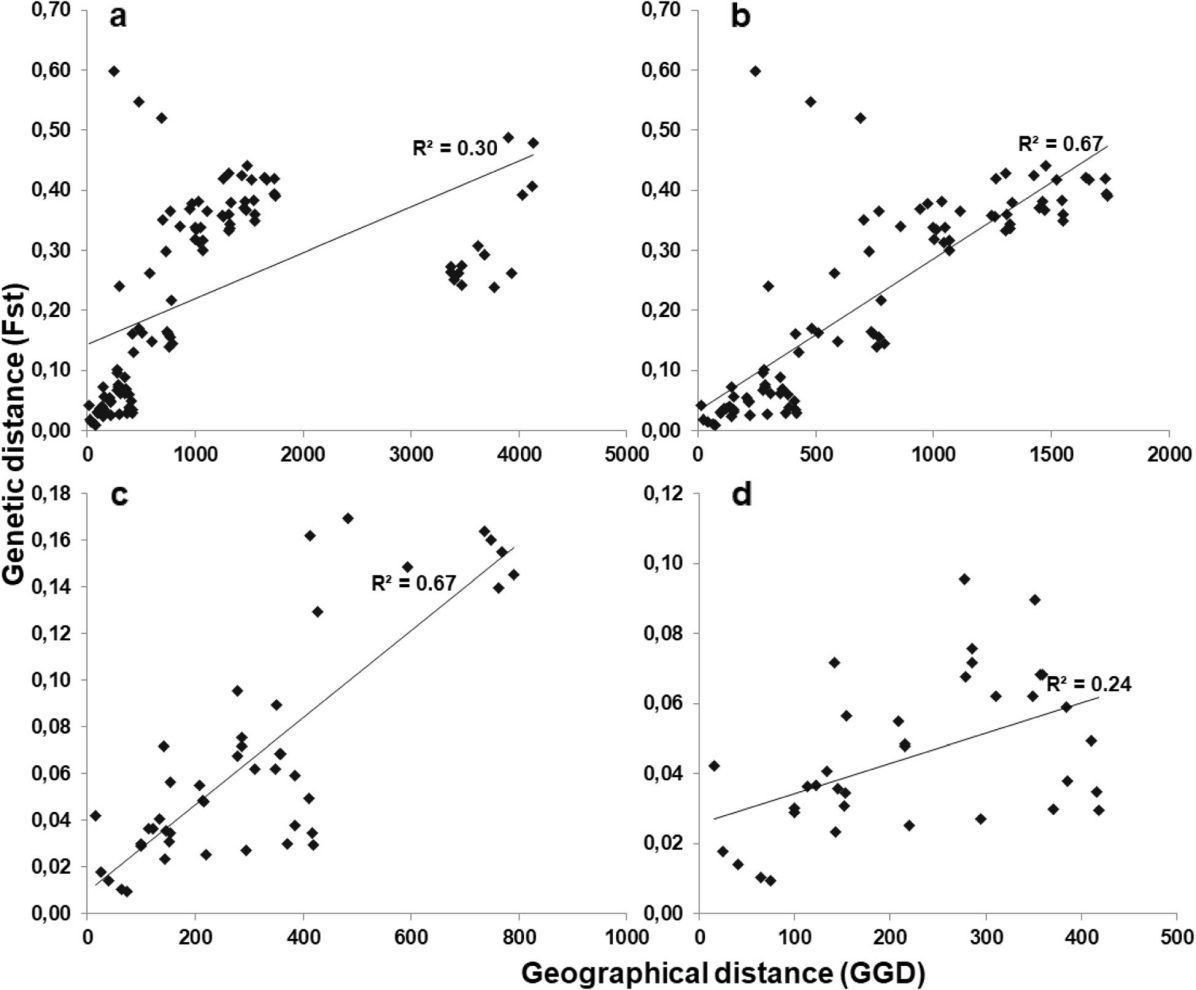
Mantel tests for correlations between genetic distance (F_st_) and Euclidean geographical distance (GGD in Km) for *O. niloticus* populations. **a** represents isolation by distance (IBD) between all populations, **b** all populations without Burkina Faso, **c** East African, and **d** only Ugandan populations

### Genotyping

Genomic DNA extraction was conducted using magnetic beads based on the MagSi-DNA Vegetal kit (MagnaMedics, Geleen, Netherlands) and a magnetic separator, SL-MagSep96 (Steinbrenner, Germany) [27, 30]. We used microsatellite markers [27], to which we added 15 extra primers (Table 2, see also Additional file 1: Table S3). The SSR primers were designed and tested following our earlier work [27], using the same shotgun sequencing data present in the sequence read archive database (SRA) under the reference number SRX3398501. Screened primers were then grouped into three multiplexes and used to prepare amplicon SSR-GBS libraries using the same approach and specifications of [27]. The PCR products were then pooled and sent for paired-end 300 bp sequencing in Illumina MiSeq, at the Genomics Service Unit in Ludwig Maximillian Universität, München, Germany. The raw sequence data were deposited in the GenBank, sequence read archive database (SRA) under the project PRJNA550300 with the accession numbers, SRR9587388 to SRR9587270. Sequences generated by Illumina, were subsequently quality checked and controlled, which were later used for alleles calling as described in [27, 30] using the scripts from the SSR-GBS pipeline (https://github.com/mcurto/SSR-GBS-pipeline). The resulting codominant matrix and information for which sequences correspond to each allele can be found in the Additional file 2 (see the file named “Second_additional fileAllelesList & matrix_”).For subsequent analyses, all loci and samples with missing genotypes ≥50% were excluded, leaving a total number of 40 markers (Additional file 1: Tables S1, S3). Other studies have indicated that many SSR loci are not necessary in order to detect population structure [55, 56], so we did not see the need of developing additional markers to the 40 already in use.

**Table 2:** 15 new primer pairs developed in the present study. The other 26 tested primers developed by [27] can be found in the additional file section, Additional file 1: Table S1

Locus	F: Primer sequence (5′-3′)	R: Primer sequence (5′-3′)	Repeat motif	Asr
Ti39	TACCTGCCAGTCATGTGCTG	TGCTCAGACTGGTCCCTTCT	(ATGG)8	368–420
Ti41	TCGCAGCTGCTCCTGTTTAA	TTGTGCACGTGGACATGTTG	(AAAC)11	381–471
Ti43	ATTGCCATCACCAGGAACCA	TGCTAGCCCAGAGCATTTGA	(GAATA)6	425–478
Ti44	TGCTCCTGACTCAGCATCAC	GCAGCACTCTGACATGAAGC	(GAAAA)6	419–469
Ti49	TCGAAGTAGCGTGGAAAACCT	ACAACAACAACAGGTCGGGA	(TGT)8	395–403
Ti50	CCTGTGACAGACTGGTGACC	ACACTGATGCGGTTTACGGT	(ATGG)7	442–517
Ti51	TGCTAAACGCCAGCTGATGA	TTACCACACGATGTCGCAGG	(TGT)8	401–428
Ti52	GAGAAACGTCCAGTGGCAGA	TTTCGATCTGCTGCCCCTTT	(TAT)8	373–429
Ti54	TTTCTTGCCAGCAAAAACAGT	CAGATTCTTCCAGTGCTTGTGC	(GGAT)7	390–480
Ti55	GAGCCCAGACAGCAGACAAT	AGGACCTTCTATGGCCCTGT	(TCTA)7	417–491
Ti56	TGCAGTGAATTTGGCACCTG	AGCCTGAGATACCTGTGCCT	(TGTT)6	310–462
Ti57	CAGTGGGAGGAAGCTCCAAA	GCTGCATGGATCCAATAGGC	(TCCA)7	400–444
Ti59	ATGGACTTAAGCTGCACCCC	TGAGCATTTGACCCCAGCAT	(AGGA)6	429–461
Ti60	GAGCCGCCATAGTGTCACTT	CCTGCTCTCACTCAAAGAGGG	(ATCC)7	473–516
Ti61	GCTACACAGGAAAGCAGAGC	ACTCAATGCTGGACGTGACC	(TGGA)6	474–501

*F* Forward and *R* Reverse, *Asr* Allelic size range.

### Genetic structure

Genetic structure was first assessed by calculating the genetic distance between individuals and then visualized through Principal Coordinate Analysis (PCoA), all conducted in GenAlex Version 6.5 [57]. Genetic similarity between populations was evaluated by plotting a Neighbor-Net tree based on Nei’s genetic distance [58], using the program, SplitsTree4 version, 4.14.8 [59]. We also constructed UPGMA dendrograms for making inferences on the hierarchical clustering using Nei’s genetic distance as implemented in Populations-1.2.32 [60]. Support values were estimated with 1000 bootstrap replicates based on loci resampling. Neighbor-Net tree and the UPGMA dendrogram were conducted with the inclusion of subpopulations, when applicable to evaluate possible substructure within the populations. Genetic structure was further investigated using the program, STRUCTURE Version 2.3.4 [61]. STRUCTURE clusters individuals into hypothetical populations through optimization of Hardy-Weinberg equilibrium [62]. STRUCTURE was run from K = 1–35 for 10,000 Markov chain Monte Carlo (MCMC) generations after a burn-in length of 10,000 generations [63], whereby each run was iterated 20 times. The program’s default settings for the admixture model and allele frequencies correlated were implemented. Detection of optimal K was done with STRUCTURE HARVESTER [64] using the delta K (ΔK) statistic, which is the second-order rate of change (InP(D)) across successive K values [63, 65]. In this context, STRUCTURE HARVESTER uses ΔK to identify the highest value and henceforth the best K. Results from multiple replicates were summarized using the online pipeline Clumpak program [66] available at http://clumpak.tau.ac.il/. Similar analyses were performed for Lake Victoria within populations.

### Migration rates and number of migrants per generation (nm)

Recent migratory rates and the number of migrants per generation were determined as proxy estimates of gene flow among the *O. niloticus* populations. However, recent migratory rates were only estimated for the East African populations, since the corresponding water bodies are the most affected by anthropogenic activities such as fish translocations. Pairwise recent migration rates were estimated using BayesAss Version 3.0 [67]. Here, the program was run for 200, 000,000 iterations, discarding the first 100,000,000 generations and sampling every 1000th generation [68]. Only results with a 95% confidence interval of a fraction of migrants per population above 0.01 were considered significant. Recent migration rates were used because most of the fish translocations in the region, seemingly were recent. Additionally, we estimated the number of migrants (Nm) per generation between population pairs, to validate the recent migration rates using GenAlex program. Consequently, we present both, the percentage of migrants estimated in BayesAss and the number of migrants between population pairs against the fixation index (F_st_) values.

### Genetic diversity, differentiation, and isolation by distance (IBD)

Genetic diversity and differentiation indices between *O. niloticus* populations throughout East Africa and beyond were examined using the following indices: expected heterozygosity (He), observed heterozygosity (Ho), number of alleles (Na), allelic richness (Ar), fixation index (F_st_), private alleles, and Garza-Williamson index (G-W). Na, F_st_, G-W and He per population were analyzed using the program Arlequin Version 3.5 [69]. Ho, He, Na and PIC per locus were determined through Cervus version 3.0.7 [70]. Ar was analyzed using the rarefaction algorithm implemented in the Hp-rare program [71]. G-W was used to explore the possibility of bottlenecks amongst the populations. If G-W values are closer to zero, it implies that the populations went through a bottleneck, but when the values are close to one, the populations are in a stable phase [72]. To test whether the genetic diversity and differentiation of *O. niloticus* populations conform to isolation by distance (IBD), we plotted genetic distance (F_st_) against the geographical distance (GGD in kilometers) and conducted correlation analyses using Mantel test (999 permutations) implemented in GenAlex Version 6.5 [57].

## Supplementary information

**Additional file 1: Table S1.** Final List of 40 SSR primers utilized in PCR reactions. **Table S2.** Variation of the final set of 40 SSR loci. Loci Ti1–Ti35 were taken from Tibihika et al. [28] while loci Ti39–Ti61 were developed in the current study. **Table S3.** List of the linkage group and location/position for each of the primer pairs used. These primers were BLAST against the genome assembly GCF_001858045.2 available in GenBank. The field reference refers to the accession number of the matching Linkage group. **Figure S1.** Genetic structure based on PCoA within the East African Western Rift Valley lakes, Albert (**a**) and Edward (**b**) metapopulations. **Figure S2.** STRUCTURE HARVESTER analyses for depicting the optimal K values, which is derived from STRUCTURE results. **a** represents the best K for all populations (K = 10), **b** best K for all native populations (K = 7), **c** best K for East African native populations (K = 2), **d** best K for only Ugandan native populations (K = 2), **e** best K for all Ugandan populations including farms (K = 4) and **f** best K for Lake Victoria within populations (K = 2). **Figure S3.** Bayesian clustering for genetic assignments of *O. niloticus* populations. **a** represents East African native, **b** Only Uganda native populations and **c** Lake Victoria subpopulations. K values with a superscript symbol (¶) indicate the optimal K value based on STRUCTURE HARVESTER analyses. **Table S3.** Matrix for the number of migrants per generation between population pairs as evidenced by Fst values. Bold values (> 10) indicate the vital number of migrants explained by Fst values: Note the variations in boldness which explains the level of important migrations between population pairs. **Figure S4.** Within-population genetic diversity of Albert, Edward, Kyoga, and Victoria populations. Vic = Victoria, Na = mean number of alleles, No. = number, Pa = mean number of private alleles and He = mean expected heterozygosity.

**Additional file 2.** Allele list.

## Data Availability

Raw sequence data were submitted to the sequence read archive (SRA) database and can be accessed under the reference number PRJNA550300.

## Abbreviations

Ar: Allelic richness
G-W: Garza-Williamson index
GenAlex: Genetic Analysis in excel
He: expected heterozygosity
Ho: observed heterozygosity
IBD: Isolation By Distance
MCMC: Markov chain Monte Carlo
mtDNA: mitochondrial DNA
Na: Number of alleles
PCoA: Principal Coordinates Analysis
PIC: Polymorphic Information Content
RAPD: Randomly Amplified Polymorphic DNA
RFLP: Restriction FragmentLlength Polymorphism
SRA: Sequence Read Archive
SSR-GBS: Simple Sequence Repeat- Genotyping By Sequencing
SSRs: Simple Sequence Repeats
UPGMA: Unweighted Pair Group Method with Arithmetic Mean
