# Supplementary material for: Molecular genetic diversity and differentiation of Nile tilapia (Oreochromis niloticus, L. 1758) in East African natural and stocked populations
Source: BMC Evol Biol. 2020 Jan 30;20:16. doi: 10.1186/s12862-020-1583-0 (PMC6990601; doi:10.1186/s12862-020-1583-0)
Supplement: Supplementary file 1 — Additional file 1: Table S1. Final List of 40 SSR primers utilized in PCR reactions. Table S2. Variation of the final set of 40 SSR loci. Loci Ti1–Ti35 were taken from Tibihika et al. [28] while loci Ti39–Ti61 were developed in the current study. Table S3. List of the linkage group and location/position for each of the primer pairs used. These primers were BLAST against the genome assembly GCF_001858045.2 available in GenBank. The field reference refers to the accession number of the matching Linkage group. Figure S1. Genetic structure based on PCoA within the East African Western Rift Valley lakes, Albert (a) and Edward (b) metapopulations. Figure S2. STRUCTURE HARVESTER analyses for depicting the optimal K values, which is derived from STRUCTURE results. a represents the best K for all populations (K = 10), b best K for all native populations (K = 7), c best K for East African native populations (K = 2), d best K for only Ugandan native populations (K = 2), e best K for all Ugandan populations including farms (K = 4) and f best K for Lake Victoria within populations (K = 2). Figure S3. Bayesian clustering for genetic assignments of O. niloticus populations. a represents East African native, b Only Uganda native populations and c Lake Victoria subpopulations. K values with a superscript symbol (¶) indicate the optimal K value based on STRUCTURE HARVESTER analyses. Table S3. Matrix for the number of migrants per generation between population pairs as evidenced by Fst values. Bold values (> 10) indicate the vital number of migrants explained by Fst values: Note the variations in boldness which explains the level of important migrations between population pairs. Figure S4. Within-population genetic diversity of Albert, Edward, Kyoga, and Victoria populations. Vic = Victoria, Na = mean number of alleles, No. = number, Pa = mean number of private alleles and He = mean expected heterozygosity. [file 12862_2020_1583_MOESM1_ESM.docx]

**Molecular genetic diversity and differentiation of Nile tilapia (*Oreochromis niloticus*, L. 1758) in East African natural and stocked populations**

Papius Dias Tibihika^†, §^, Manuel Curto^†^, Esayas Alemayehu^‡, ß^, Herwig Waidbacher^‡^, Peter Akoll^¶^, Charles Masembe^¶^, Harald Meimberg^†^

^†^ Institute for Integrative Nature Conservation Research, University of Natural Resources and Life Sciences Vienna (BOKU), Gregor Mendel Straße 33, 1180 Wien, Austria. [papius.tibihika@students.boku.ac.at](about:blank), [manuel.curto@boku.ac.at](about:blank), [meimberg@boku.ac.at](about:blank)

^‡^ Institute for Hydrobiology and Aquatic Ecosystems Management, University of Natural Resources and Life Sciences Vienna (BOKU), Gregor Mendel Straße 33/DG, 1180 Wien, Austria. [esayas.negash@students.boku.ac.at](about:blank), [herwig.waidbacher@boku.ac.at](about:blank)

^§^ National Agricultural Research Organization, Kachwekano Zonal Agricultural Research and Development Institute, P.O. Box 421, Kabale, Uganda. [papiust@yahoo.com](about:blank)

^¶^ Department of Zoology, Entomology and Fisheries Sciences-Makerere University Kampala, Uganda, P. O. Box 7062, Kampala. [pakoll@cns.mak.ac.ug](about:blank), [cmasembe@cns.mak.ac.ug](about:blank)

^ß^ National Fishery and Aquatic Life Research Centre, Sebeta, P.O. Box 64, Addis Ababa, Ethiopia. [esayas.negash@students.boku.ac.at](about:blank)

^§^ Corresponding author; Papius Dias Tibihika

papiust@yahoo.com, papius.tibihika@students.boku.ac.at

Tel. +256 772 684 770

**Additional file 1**

**Table S1** Final List of 40 SSR primers utilized in PCR reactions

| **Locus** | **F: Primer sequence (5'-3')** | **R: Primer sequence (5'-3')** | **Repeat motif** | **Asr** |
| --- | --- | --- | --- | --- |
| Ti1 | TTATCACTGCTGAACGTCTT | GTTTTGGCTGCTACACATTC | (TG)10 | 393-411 |
| Ti2 | TTCTGGGCTAACACACAAG | AAGGTGTCACACAGTTTAGG | (CA)18 | 396-438 |
| Ti4 | TGTGCAGAATAGAATAGCCC | GAAAGGAAAAATGTTGGTGGT | (GT)18 | 384-436 |
| Ti5 | AAGGAGGATGATCAGGACAC | AGACCTCCACTGTGATCTTA | (CA)10 | 401-423 |
| Ti6 | CAGCTCTCATGAACACTTGA | ACCCATAAATCACACCAGTC | (GA)23 | 384-437 |
| Ti7 | TCTTTGTGTCAGAACTGTGT | ACTCTGCTTTTAGCCAATCA | (AC)17 | 385-430 |
| Ti8 | CTGAAGTCCTGCTGAGATTT | CATTGTTCTTGGCACCTCTA | (AC)15 | 400-446 |
| Ti9 | CTCAGTGACGAAGCCAAA | CCTGGCAATCAAAAGAACAA | (AC)10 | 409-419 |
| Ti12 | GCCACCAAAATATTCGTGTT | CCATGTTCTGTCTCCTTGAA | (TAC)12 | 377-431 |
| Ti13 | AATCCGTTAGCTGCAGATAG | GCTGATTAAACACAAAGTTGG | (ATG)10 | 369-427 |
| Ti14 | TCCCTAAAATATGCCACCAA | TAGTGCTTTAATGGCTCTGG | (TAA)19 | 340-408 |
| Ti15 | GCTGTGATCATCTGGAGAAA | AGGATCTAGAACCTCCAACC | (TGC)10 | 385-406 |
| Ti16 | CAGACGTAGGCGATAAATCT | GAACACATCCATTTCCACAC | (AAC)10 | 368-428 |
| Ti17 | AACTGAAGAAGAAGCCTTGG | ATCATCTTCCTCTACTGCCT | (GAA)21 | 361-430 |
| Ti18 | AGCAAGTGAGATAAGCACTG | TACATAGCAGTGCAGTTTGC | (ATCT)8 | 373-461 |
| Ti24 | ACTGACAACATAAAGACATATGC | CACAGTTTGAATCCACCATC | (TTAC)9 | 403-483 |
| Ti26 | ATTGCTTCATCCCTTGAGTT | ACACGGAAAACCTAATGACA | (ACAA)10 | 402-475 |
| Ti27 | CTGTCTTTCTTGATGTGGGA | ATGCACAAATTTTAAGGGCC | (TTTG)6 | 391-405 |
| Ti28 | TGTCTTGGGATTTGAGATCA | CGGAGGTTTCTTCCTGTTAA | (ATTCA)8 | 307-409 |
| Ti29 | AGGTCAAGATCAAGCAGTTT | CATCAACATAATTCAGTGTGGA | (TAAAA)10 | 321-400 |
| Ti31 | GAAACTATCCACAGAAGCCA | AGGCTTCTTACAGTTGGATG | (CTAAT)7 | 302-395 |
| Ti32 | CAGGAAATGGCTCCAAAATG | TTGTAGCTAGGAATCAGTGC | (AAAAT)7 | 325-361 |
| Ti33 | GCTTATGGCTGTATGGAGTT | CGACTTCTGTTGTGATTTGG | (TTCAA)6 | 382-427 |
| Ti34 | GCTTACAGTACATTGTGTGC | CTGATGAGAAAAACAGACGC | (TCTCT)10 | 355-448 |
| Ti35 | TCAACCACAAACTCCTCTTT | AAACTAAGTGCAGCTCATGA | (AAAAG)14 | 364-434 |
| Ti39 | TACCTGCCAGTCATGTGCTG | TGCTCAGACTGGTCCCTTCT | (ATGG)8 | 368-420 |
| Ti41 | TCGCAGCTGCTCCTGTTTAA | TTGTGCACGTGGACATGTTG | (AAAC)11 | 381-471 |
| Ti43 | ATTGCCATCACCAGGAACCA | TGCTAGCCCAGAGCATTTGA | (GAATA)6 | 425-478 |
| Ti44 | TGCTCCTGACTCAGCATCAC | GCAGCACTCTGACATGAAGC | (GAAAA)6 | 419-469 |
| Ti49 | TCGAAGTAGCGTGGAAAACCT | ACAACAACAACAGGTCGGGA | (TGT)8 | 395-403 |
| Ti50 | CCTGTGACAGACTGGTGACC | ACACTGATGCGGTTTACGGT | (ATGG)7 | 442-517 |
| Ti51 | TGCTAAACGCCAGCTGATGA | TTACCACACGATGTCGCAGG | (TGT)8 | 401-428 |
| Ti52 | GAGAAACGTCCAGTGGCAGA | TTTCGATCTGCTGCCCCTTT | (TAT)8 | 373-429 |
| Ti54 | TTTCTTGCCAGCAAAAACAGT | CAGATTCTTCCAGTGCTTGTGC | (GGAT)7 | 390-480 |
| Ti55 | GAGCCCAGACAGCAGACAAT | AGGACCTTCTATGGCCCTGT | (TCTA)7 | 417-491 |
| Ti56 | TGCAGTGAATTTGGCACCTG | AGCCTGAGATACCTGTGCCT | (TGTT)6 | 310-462 |
| Ti57 | CAGTGGGAGGAAGCTCCAAA | GCTGCATGGATCCAATAGGC | (TCCA)7 | 400-444 |
| Ti59 | ATGGACTTAAGCTGCACCCC | TGAGCATTTGACCCCAGCAT | (AGGA)6 | 429-461 |
| Ti60 | GAGCCGCCATAGTGTCACTT | CCTGCTCTCACTCAAAGAGGG | (ATCC)7 | 473-516 |
| Ti61 | GCTACACAGGAAAGCAGAGC | ACTCAATGCTGGACGTGACC | (TGGA)6 | 474-501 |

F=Forward and R=Reverse, Asr=Allelic size range

**Table S2** Variation of the final set of 40 SSR loci. Loci Ti1–Ti35 were taken from Tibihika et al. (28) while loci Ti39–Ti61 were developed in the current study

| **Loci** | **Na** | **Ho** | **He** | **Ht** | **PIC** |
| --- | --- | --- | --- | --- | --- |
| Ti1 | 17 | 0.377 | 0.571 | 0.601 | 0.500 |
| Ti2 | 54 | 0.704 | 0.854 | 0.864 | 0.844 |
| Ti4 | 22 | 0.757 | 0.842 | 0.862 | 0.823 |
| Ti5 | 19 | 0.438 | 0.644 | 0.671 | 0.614 |
| Ti6 | 50 | 0.866 | 0.841 | 0.879 | 0.828 |
| Ti7 | 35 | 0.843 | 0.820 | 0.833 | 0.801 |
| Ti8 | 29 | 0.197 | 0.387 | 0.469 | 0.375 |
| Ti9 | 10 | 0.156 | 0.597 | 0.589 | 0.558 |
| Ti12 | 36 | 0.306 | 0.552 | 0.633 | 0.527 |
| Ti13 | 12 | 0.224 | 0.449 | 0.485 | 0.431 |
| Ti14 | 18 | 0.341 | 0.618 | 0.708 | 0.591 |
| Ti15 | 14 | 0.138 | 0.417 | 0.461 | 0.381 |
| Ti16 | 30 | 0.126 | 0.423 | 0.507 | 0.407 |
| Ti17 | 64 | 0.661 | 0.820 | 0.861 | 0.804 |
| Ti18 | 76 | 0.652 | 0.951 | 0.950 | 0.948 |
| Ti24 | 68 | 0.654 | 0.879 | 0.900 | 0.873 |
| Ti26 | 20 | 0.480 | 0.735 | 0.734 | 0.696 |
| Ti27 | 7 | 0.060 | 0.325 | 0.390 | 0.310 |
| Ti28 | 15 | 0.201 | 0.386 | 0.420 | 0.361 |
| Ti29 | 39 | 0.230 | 0.718 | 0.808 | 0.675 |
| Ti31 | 24 | 0.191 | 0.830 | 0.828 | 0.811 |
| Ti32 | 84 | 0.938 | 0.931 | 0.929 | 0.926 |
| Ti33 | 18 | 0.606 | 0.832 | 0.823 | 0.809 |
| Ti34 | 79 | 0.811 | 0.940 | 0.946 | 0.936 |
| Ti35 | 56 | 0.562 | 0.951 | 0.951 | 0.948 |
| Ti39 | 33 | 0.384 | 0.644 | 0.686 | 0.615 |
| Ti41 | 50 | 0.539 | 0.880 | 0.904 | 0.871 |
| Ti43 | 61 | 0.364 | 0.654 | 0.729 | 0.647 |
| Ti44 | 29 | 0.600 | 0.839 | 0.850 | 0.821 |
| Ti49 | 24 | 0.303 | 0.381 | 0.427 | 0.370 |
| Ti50 | 22 | 0.434 | 0.616 | 0.695 | 0.584 |
| Ti51 | 40 | 0.545 | 0.759 | 0.789 | 0.737 |
| Ti52 | 14 | 0.271 | 0.694 | 0.776 | 0.651 |
| Ti54 | 47 | 0.677 | 0.920 | 0.932 | 0.914 |
| Ti55 | 29 | 0.728 | 0.905 | 0.902 | 0.897 |
| Ti56 | 24 | 0.200 | 0.747 | 0.784 | 0.721 |
| Ti57 | 16 | 0.054 | 0.401 | 0.476 | 0.370 |
| Ti59 | 28 | 0.596 | 0.803 | 0.835 | 0.778 |
| Ti60 | 22 | 0.531 | 0.737 | 0.740 | 0.697 |
| Ti61 | 17 | 0.127 | 0.259 | 0.307 | 0.250 |
| **Total** | **1,352** |  | **80%>0.5** | **82.5>0.5** | **78%>0.5** |

Na = number of alleles, Ho = observed heterozygosity, He = expected heterozygosity, Ht = total expected heterozygosity, PIC = Polymorphic Information Content.

**Table S3** List of the linkage group and location/position for each of the primer pairs used. These primers were BLAST against the genome assembly GCF_001858045.2 available in GenBank. The field reference refers to the accession number of the matching Linkage group.

| **Marker** | **Linkage group** | **Reference** | **Position Forward** | **Position reverse** |
| --- | --- | --- | --- | --- |
| TI1 | LG8 | NC_031973.2 | 24710793 | 24710395 |
| TI2 | LG14 | NC_031979.2 | 13863615 | 13864024 |
| TI4 | LG6 | NC_031971.2 | 33952328 | 33952740 |
| TI5 | LG19 | NC_031983.2 | 9336428 | 9336840 |
| TI6 | LG17 | NC_031981.2 | 28396929 | 28397334 |
| TI7 | LG12 | NC_031977.2 | 18222355 | 18222774 |
| TI8 | LG23 | NC_031986.2 | 24646382 | 24646795 |
| TI9 | LG13 | NC_031978.2 | 17954553 | 17954965 |
| TI12 | LG11 | NC_031976.2 | 18705528 | 18705152 |
| TI13 | LG5 | NC_031970.2 | 34236443 | 34236041 |
| TI14 | LG4 | NC_031969.2 | 21218236 | 21218603 |
| TI15 | LG23 | NC_031986.2 | 42756320 | 42756716 |
| TI16 | LG4 | NC_031969.2 | 21361446 | 21361058 |
| TI17 | LG20 | NC_031984.2 | 11840889 | 11840508 |
| TI18 | LG23 | NC_031986.2 | 7360986 | 7361405 |
| TI22 | LG3 | NC_031967.2 | 22099501 | 22099151 |
| TI24 | LG13 | NC_031978.2 | 15154003 | 15153601 |
| TI26 | LG7 | NC_031972.2 | 26690324 | 26690734 |
| TI27 | LG7 | NC_031972.2 | 59798822 | 59798432 |
| TI28 | LG15 | NC_031980.2 | 27884489 | 27884106 |
| TI29 | LG20 | NC_031984.2 | 19328265 | 19328644 |
| TI31 | LG16 | NC_031987.2 | 19763717 | 19764076 |
| TI32 | LG12 | NC_031977.2 | 9749037 | 9749381 |
| TI33 | LG16 | NC_031987.2 | 5908000 | 5908352 |
| TI34 | LG12 | NC_031977.2 | 15764851 | 15765215 |
| TI35 | LG18 | NC_031982.2 | 13056722 | 13057115 |
| TI36 | LG14 | NC_031979.2 | 39677178 | 39677612 |
| TI37 | LG22 | NC_031985.2 | 25336906 | 25336501 |
| TI38 | LG1 | NC_031965.2 | 23203642 | 23204154 |
| TI39 | LG13 | NC_031978.2 | 27572416 | 27572021 |
| TI41 | LG17 | NC_031981.2 | 25962525 | 25962951 |
| TI43 | LG14 | NC_031979.2 | 24278031 | 24277607 |
| TI44 | LG16 | NC_031987.2 | 28045194 | 28045652 |
| TI45 | LG20 | NC_031984.2 | 27248328 | 27247954 |
| TI46 | LG1 | NC_031965.2 | 23203642 | 23204154 |
| TI49 | LG7 | NC_031972.2 | 26681220 | 26680822 |
| TI50 | LG17 | NC_031981.2 | 30330112 | 30329604 |
| TI51 | LG2 | NC_031966.2 | 18627211 | 18626802 |
| TI52 | LG20 | NC_031984.2 | 30411415 | 30411003 |
| TI53 | LG3 | NC_031967.2 | 35054978 | 35055360 |
| TI54 | LG19 | NC_031983.2 | 23052189 | 23051762 |
| TI55 | LG14 | NC_031979.2 | 3705138 | 3705572 |
| TI56 | LG17 | NC_031981.2 | 5401996 | 5401547 |
| TI57 | LG1 | NC_031965.2 | 16299520 | 16299121 |
| TI59 | LG4 | NC_031969.2 | 33210178 | 33210606 |
| TI60 | LG18 | NC_031982.2 | 5638757 | 5638270 |
| TI61 | LG6 | NC_031971.2 | 27860186 | 27860681 |


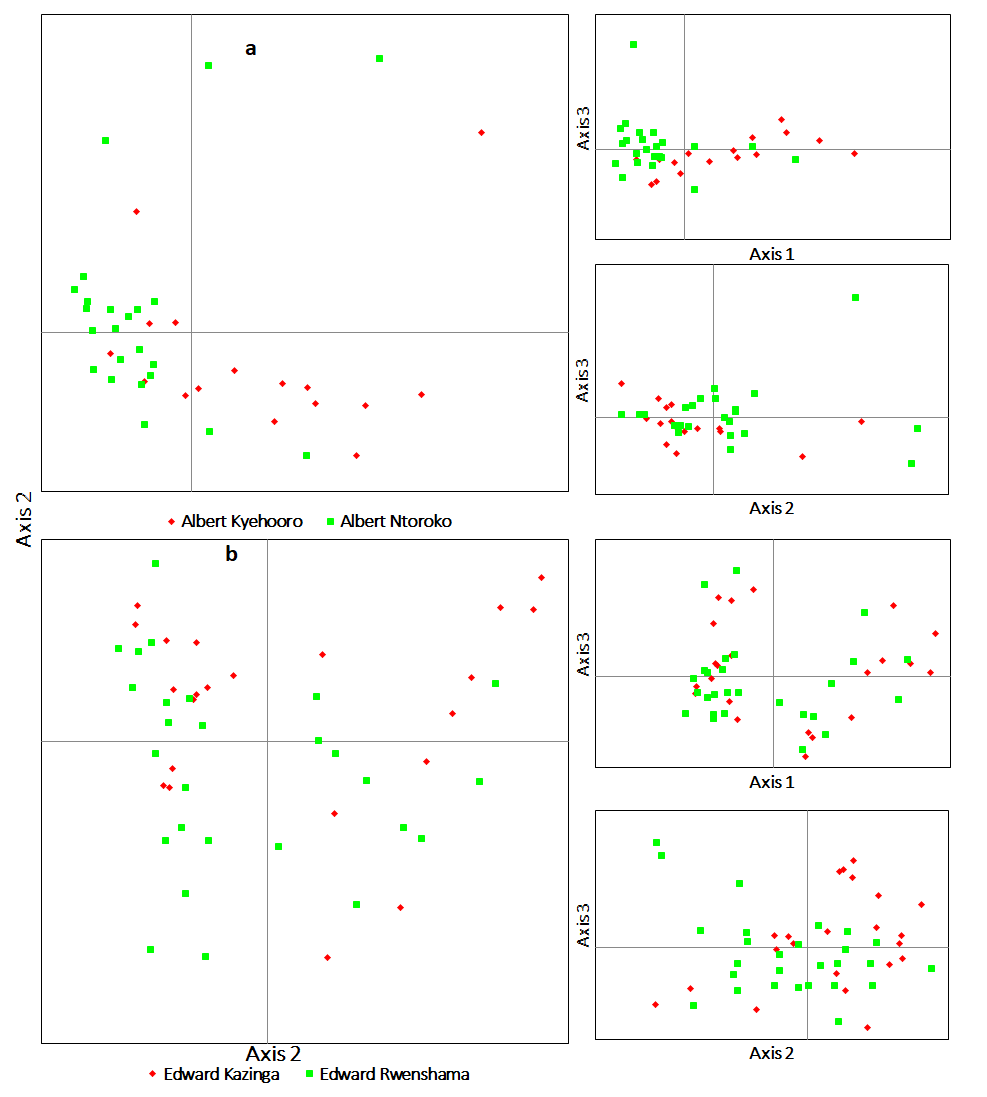


**Figure S1** Genetic structure based on PCoA within the East African Western Rift Valley lakes, Albert (**a**) and Edward (**b**) metapopulations

**
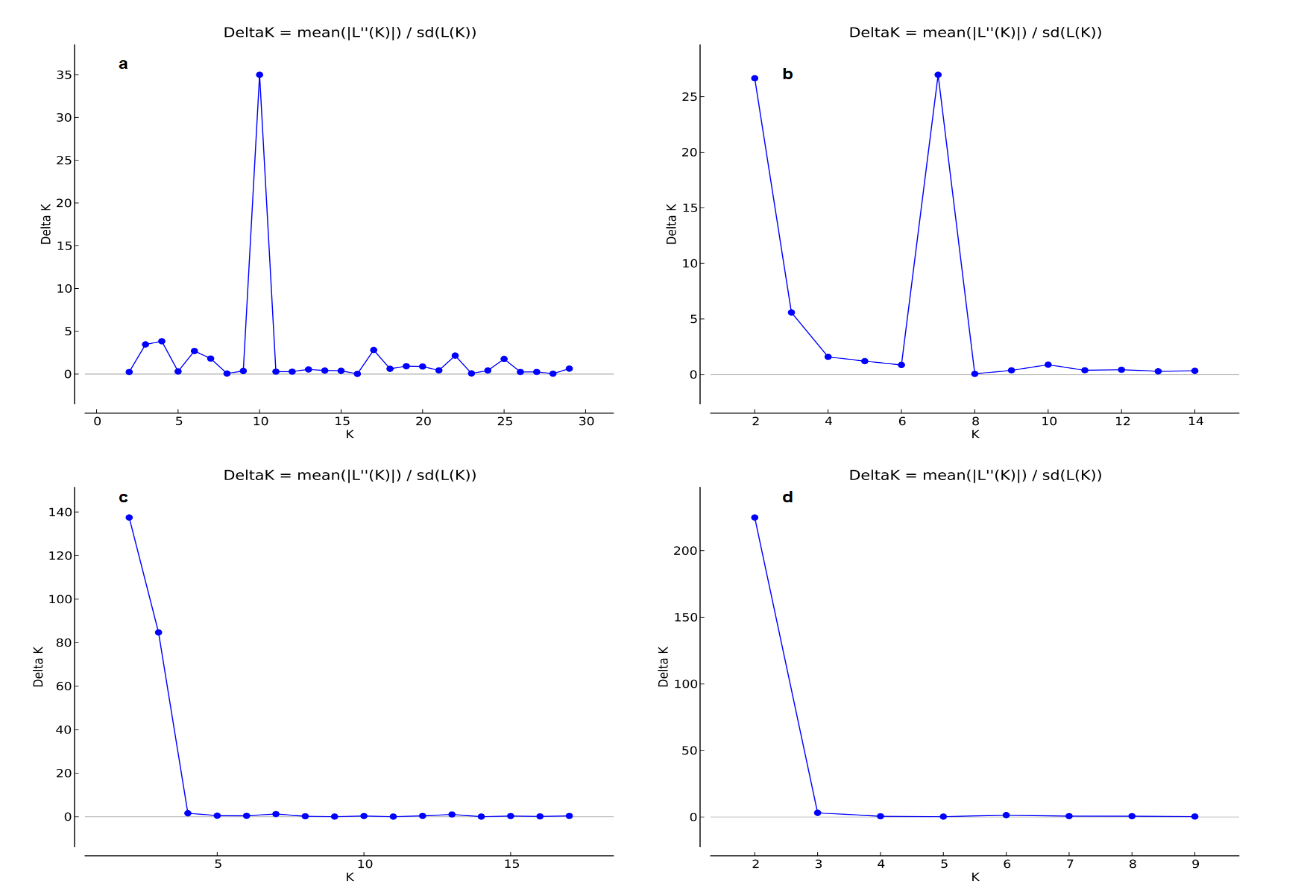
**

**
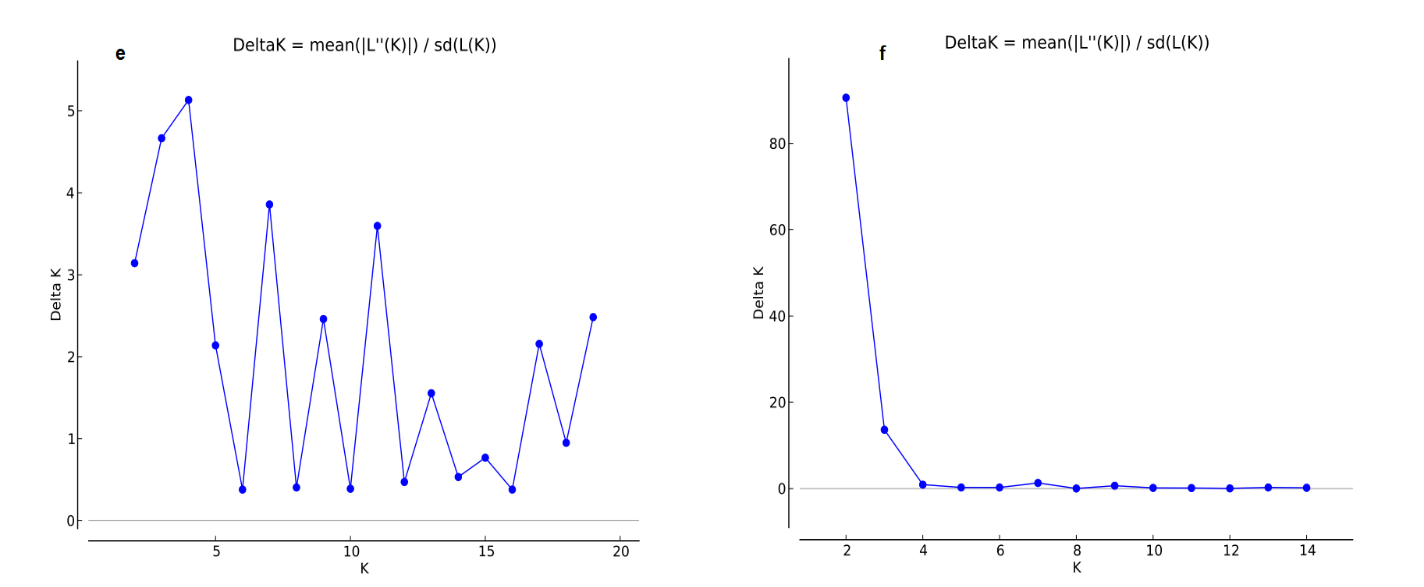
**

**Figure S2** STRUCTURE HARVESTER analyses for depicting the optimal K values, which is derived from STRUCTURE results. **a** represents the best K for all populations (K=10), **b** best K for all native populations (K=7), **c** best K for East African native populations (K=2), **d** best K for only Ugandan native populations (K=2), **e** best K for all Ugandan populations including farms (K=4) and **f** best K for Lake Victoria within populations (K=2)


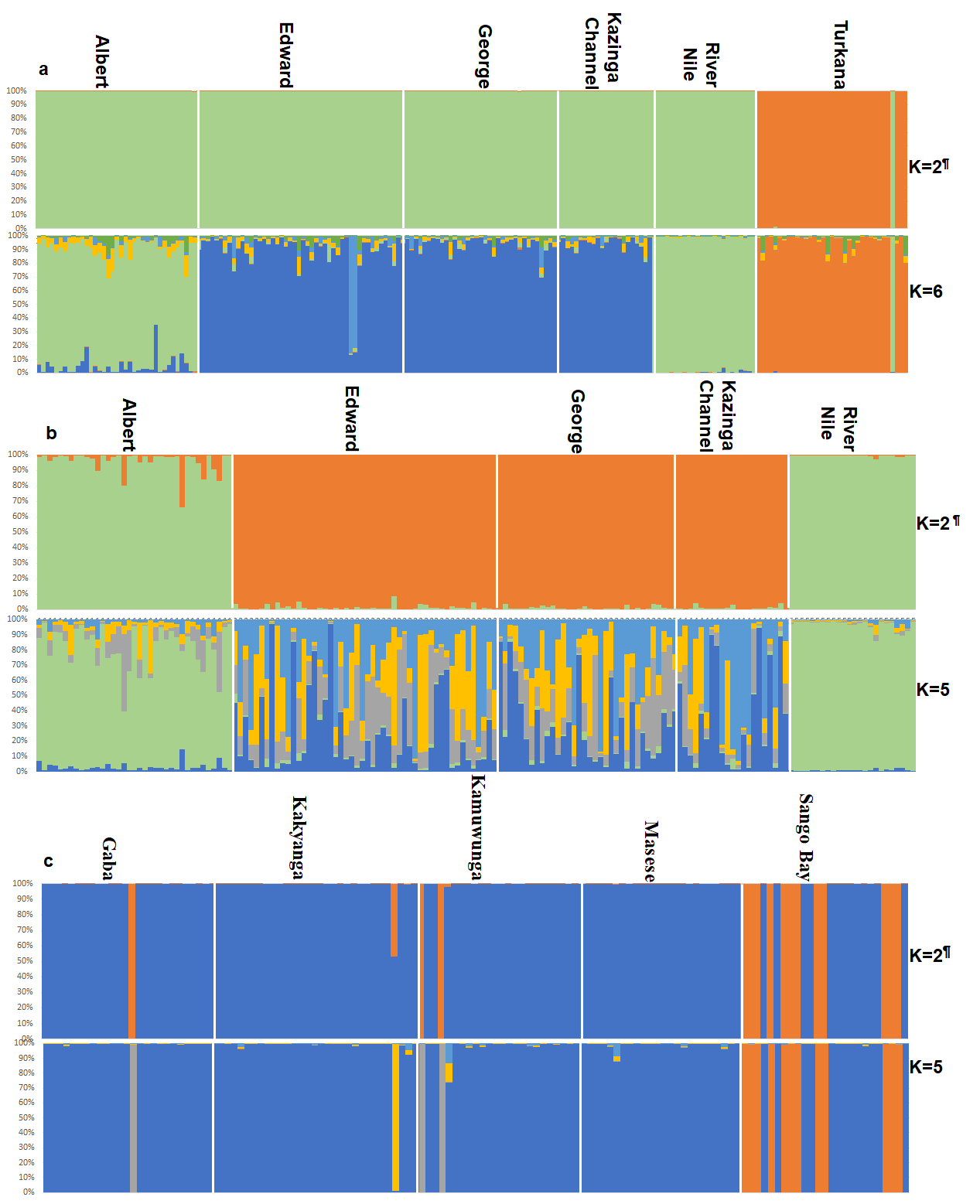


**Figure S3** Bayesian clustering for genetic assignments of *O. niloticus* populations. **a** represents East African native, **b** Only Uganda native populations and **c** Lake Victoria subpopulations. K values with a superscript symbol (¶) indicate the optimal K value based on STRUCTURE HARVESTER analyses.

**Table S3** Matrix for the number of migrants per generation between population pairs as evidenced by Fst values. Bold values (>10) indicate the vital number of migrants explained by Fst values: Note the variations in boldness which explains the level of important migrations between population pairs

| Population pair |  | Fst | Nm |
| --- | --- | --- | --- |
| River Nile | Kyoga | **0.009** | **26.495** |
| Edward | George | **0.010** | **23.754** |
| Edward | Kazinga Channel | **0.014** | **17.627** |
| Kyoga | Sindi Farm | **0.017** | **14.628** |
| Albert | Bagena Farm | **0.017** | **14.341** |
| George | Kazinga Channel | **0.018** | **13.735** |
| Albert | Sindi Farm | **0.021** | **11.447** |
| George | Mulehe | **0.023** | **10.466** |
| River Nile | Sindi Farm | **0.025** | **9.878** |
| Albert | Kyoga | **0.025** | **9.604** |
| Bagena Farm | Sindi Farm | 0.027 | 9.178 |
| Albert | Victoria | 0.027 | 8.972 |
| Victoria | Sindi Farm | 0.028 | 8.764 |
| Albert | Rwitabingi Farm | 0.029 | 8.431 |
| Edward | Mulehe | 0.029 | 8.418 |
| Edward | Victoria | 0.029 | 8.242 |
| George | Victoria | 0.030 | 8.129 |
| Kyoga | Victoria | 0.030 | 8.034 |
| Albert | River Nile | 0.031 | 7.837 |
| Kyoga | Bagena Farm | 0.032 | 7.485 |
| Victoria | Bagena Farm | 0.034 | 7.206 |
| George | Kayumbu | 0.035 | 6.965 |
| Victoria | Mulehe | 0.035 | 6.950 |
| River Nile | Victoria | 0.036 | 6.745 |
| Edward | Kayumbu | 0.036 | 6.649 |
| Kazinga Channel | Mulehe | 0.037 | 6.581 |
| Rwitabingi Farm | Sindi Farm | 0.037 | 6.568 |
| River Nile | Bagena Farm | 0.038 | 6.398 |
| Kazinga Channel | Victoria | 0.038 | 6.319 |
| Kyoga | Rwitabingi Farm | 0.038 | 6.304 |
| Victoria | Rwitabingi Farm | 0.039 | 6.130 |
| Rwitabingi Farm | Bagena Farm | 0.040 | 5.967 |
| Kazinga Channel | Kayumbu | 0.041 | 5.904 |
| Mulehe | Kayumbu | 0.042 | 5.674 |
| River Nile | Rwitabingi Farm | 0.046 | 5.239 |
| Albert | Edward | 0.048 | 4.964 |
| Albert | George | 0.049 | 4.899 |
| Victoria | Kayumbu | 0.049 | 4.802 |
| George | Bagena Farm | 0.050 | 4.754 |
| Edward | Bagena Farm | 0.052 | 4.565 |
| George | Sindi Farm | 0.052 | 4.515 |
| Edward | Sindi Farm | 0.054 | 4.399 |
| Albert | Kazinga Channel | 0.055 | 4.288 |
| Mulehe | Bagena Farm | 0.055 | 4.266 |
| Albert | Mulehe | 0.056 | 4.185 |
| Mulehe | Sindi Farm | 0.057 | 4.160 |
| Kazinga Channel | Bagena Farm | 0.058 | 4.082 |
| Edward | Kyoga | 0.059 | 3.980 |
| Kazinga Channel | Sindi Farm | 0.061 | 3.826 |
| Edward | River Nile | 0.062 | 3.774 |
| George | Kyoga | 0.062 | 3.773 |
| Edward | Rwitabingi Farm | 0.068 | 3.444 |
| George | River Nile | 0.068 | 3.438 |
| Kazinga Channel | Kyoga | 0.068 | 3.405 |
| Kyoga | Mulehe | 0.068 | 3.405 |
| George | Rwitabingi Farm | 0.069 | 3.378 |
| Albert | Kayumbu | 0.072 | 3.243 |
| Kazinga Channel | River Nile | 0.072 | 3.232 |
| Mulehe | Rwitabingi Farm | 0.072 | 3.211 |
| Kayumbu | Bagena Farm | 0.073 | 3.184 |
| Kayumbu | Sindi Farm | 0.075 | 3.084 |
| River Nile | Mulehe | 0.076 | 3.057 |
| Kazinga Channel | Rwitabingi Farm | 0.076 | 3.035 |
| Kayumbu | Rwitabingi Farm | 0.089 | 2.561 |
| Kyoga | Kayumbu | 0.089 | 2.544 |
| River Nile | Kayumbu | 0.096 | 2.365 |
| Ziway | Chamo | 0.101 | 2.223 |
| Victoria | Turkana | 0.130 | 1.680 |
| George | Turkana | 0.140 | 1.541 |
| Edward | Turkana | 0.145 | 1.472 |
| Albert | Turkana | 0.148 | 1.435 |
| Sindi Farm | Turkana | 0.153 | 1.384 |
| Bagena Farm | Turkana | 0.153 | 1.379 |
| Kazinga Channel | Turkana | 0.155 | 1.363 |
| Rwitabingi Farm | Turkana | 0.157 | 1.342 |
| Mulehe | Turkana | 0.160 | 1.311 |
| Kyoga | Turkana | 0.162 | 1.295 |
| Ziway | Hashenge | 0.162 | 1.294 |
| Kayumbu | Turkana | 0.164 | 1.277 |
| River Nile | Turkana | 0.170 | 1.225 |
| Hashenge | Chamo | 0.217 | 0.902 |
| Victoria | Burkina Faso | 0.238 | 0.802 |
| Turkana | Chamo | 0.240 | 0.791 |
| George | Burkina Faso | 0.243 | 0.781 |
| Edward | Burkina Faso | 0.251 | 0.748 |
| Turkana | Burkina Faso | 0.261 | 0.707 |
| Kazinga Channel | Burkina Faso | 0.261 | 0.707 |
| Turkana | Ziway | 0.263 | 0.701 |
| Mulehe | Burkina Faso | 0.264 | 0.696 |
| Kayumbu | Burkina Faso | 0.273 | 0.667 |
| Albert | Burkina Faso | 0.275 | 0.659 |
| Bagena Farm | Burkina Faso | 0.279 | 0.645 |
| Sindi Farm | Burkina Faso | 0.287 | 0.620 |
| Kyoga | Burkina Faso | 0.293 | 0.603 |
| Victoria | Chamo | 0.298 | 0.590 |
| Rwitabingi Farm | Burkina Faso | 0.300 | 0.584 |
| Turkana | Hashenge | 0.300 | 0.583 |
| River Nile | Burkina Faso | 0.307 | 0.564 |
| George | Chamo | 0.312 | 0.551 |
| Edward | Chamo | 0.316 | 0.540 |
| Victoria | Ziway | 0.317 | 0.538 |
| Sindi Farm | Chamo | 0.331 | 0.506 |
| George | Ziway | 0.333 | 0.501 |
| Mulehe | Chamo | 0.335 | 0.497 |
| Edward | Ziway | 0.336 | 0.494 |
| Kazinga Channel | Chamo | 0.337 | 0.491 |
| Kayumbu | Chamo | 0.338 | 0.489 |
| Albert | Chamo | 0.339 | 0.487 |
| Victoria | Tana | 0.343 | 0.478 |
| Bagena Farm | Chamo | 0.345 | 0.475 |
| George | Tana | 0.348 | 0.469 |
| Kyoga | Chamo | 0.350 | 0.465 |
| Rwitabingi Farm | Chamo | 0.352 | 0.461 |
| Mulehe | Ziway | 0.356 | 0.452 |
| Sindi Farm | Ziway | 0.358 | 0.449 |
| Kayumbu | Ziway | 0.358 | 0.448 |
| Edward | Tana | 0.359 | 0.446 |
| Kazinga Channel | Ziway | 0.359 | 0.446 |
| Albert | Ziway | 0.364 | 0.436 |
| River Nile | Chamo | 0.364 | 0.436 |
| Victoria | Hashenge | 0.367 | 0.432 |
| Turkana | Tana | 0.368 | 0.429 |
| Bagena Farm | Ziway | 0.371 | 0.425 |
| Kayumbu | Tana | 0.371 | 0.424 |
| Kyoga | Ziway | 0.377 | 0.413 |
| Rwitabingi Farm | Ziway | 0.378 | 0.412 |
| Albert | Tana | 0.379 | 0.409 |
| Mulehe | Tana | 0.381 | 0.406 |
| Bagena Farm | Tana | 0.382 | 0.404 |
| River Nile | Ziway | 0.382 | 0.404 |
| Kazinga Channel | Tana | 0.384 | 0.401 |
| Sindi Farm | Tana | 0.387 | 0.395 |
| Edward | Hashenge | 0.391 | 0.389 |
| Chamo | Burkina Faso | 0.392 | 0.387 |
| George | Hashenge | 0.393 | 0.385 |
| Rwitabingi Farm | Tana | 0.401 | 0.374 |
| Ziway | Burkina Faso | 0.406 | 0.365 |
| Sindi Farm | Hashenge | 0.410 | 0.359 |
| Mulehe | Hashenge | 0.418 | 0.349 |
| Albert | Hashenge | 0.418 | 0.348 |
| Kazinga Channel | Hashenge | 0.419 | 0.347 |
| Kyoga | Tana | 0.420 | 0.345 |
| Kayumbu | Hashenge | 0.421 | 0.344 |
| Kyoga | Hashenge | 0.424 | 0.340 |
| Bagena Farm | Hashenge | 0.425 | 0.339 |
| Rwitabingi Farm | Hashenge | 0.426 | 0.337 |
| River Nile | Tana | 0.428 | 0.334 |
| River Nile | Hashenge | 0.441 | 0.317 |
| Hashenge | Burkina Faso | 0.480 | 0.271 |
| Tana | Burkina Faso | 0.487 | 0.263 |
| Tana | Chamo | 0.521 | 0.230 |
| Ziway | Tana | 0.548 | 0.206 |
| Tana | Hashenge | 0.599 | 0.167 |


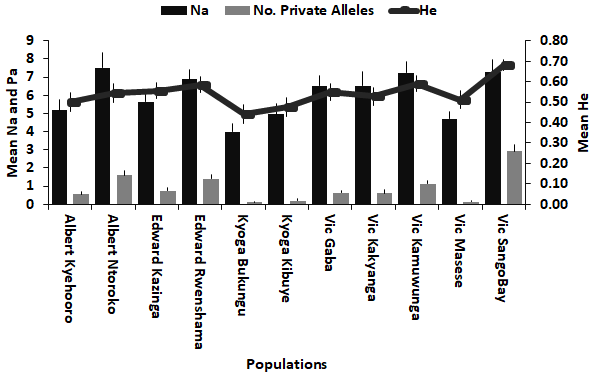


**Figure S4** Within-population genetic diversity of Albert, Edward, Kyoga, and Victoria populations. Vic=Victoria, Na=mean number of alleles, No.=number, Pa=mean number of private alleles and He=mean expected heterozygosity
